# Thymic precursor cells generate acute myeloid leukemia in NUP98-PHF23/NUP98-HOXD13 double transgenic mice

**DOI:** 10.1038/s41598-019-53610-7

**Published:** 2019-11-20

**Authors:** Subhadip Kundu, Eun Sil Park, Yang Jo Chung, Robert L. Walker, Yuelin J. Zhu, Vijay Negi, Paul S. Meltzer, Peter D. Aplan

**Affiliations:** 1 Genetics Branch, Center for cancer Research, NCI/NIH, Bethesda, MD USA; 2 Department of Pediatrics, Institute of Health Science, Gyeongsang National University College of Medicine, Gyeongsang, South Korea

**Keywords:** Acute myeloid leukaemia, Cancer genetics

## Abstract

Transgenic mice that express either a NUP98–PHF23 (NP23) or NUP98-HOXD13 (NHD13) fusion in the hematopoietic compartment develop a wide spectrum of leukemias, including myeloid, erythroid, megakaryocytic and lymphoid, at age 9–14 months. NP23-NHD13 double transgenic mice were generated by interbreeding NP23 and NHD13 mice. Remarkably, 100% of the NP23-NHD13 double transgenic mice developed acute myeloid leukemia (AML) within three months, characterized by replacement of the thymus with leukemic myeloblasts. The marked infiltration of thymus led to the intriguing hypothesis that AML generated in NP23-NHD13 mice arose in the thymus, as opposed to the bone marrow (BM). Transplantation of CD4-CD8- double negative (DN) thymocytes (which were also negative for Mac1 and Gr1) from leukemic NHD13/NP23 mice demonstrated that DN thymocytes could transmit AML, and limiting dilution studies showed that leukemia initiating cells were increased 14-fold in the thymus compared to BM. Further thymocyte fractionation demonstrated that DN1 and DN2, but not DN3 or DN4 fractions transmitted AML, and a marked expansion (100-fold) of Lineage-Sca1 + Kit + (LSK) cells in the thymus of the NP23-NHD13 mice. Taken together, these results show that the thymus of NP23-NHD13 mice acts as a reservoir for AML initiating cells and that thymic progenitors can transmit AML.

## Introduction

Hematopoietic differentiation has long been used as a paradigm for normal stem cell differentiation^1^. Through the efforts of many investigators, a hierarchy of differentiation has been established, with self-renewing, pluripotent stem cells at the apex of this hierarchy. Immediately below these pluripotent hematopoietic stem cells are committed progenitor cells, which lack infinite self-renewal potential^2^. The committed progenitors produce the mature, terminally differentiated blood cells of numerous specified lineages^3^. In adult mammals, the majority of hematopoietic differentiation, including myeloid, erythroid, megakaryocytic, and B-lymphoid differentiation is completed largely in the bone marrow, and mature blood cells are released from the bone marrow into the circulation^4^. One exception to this organization is T-lymphocyte differentiation, which takes place largely in the thymus. In the mouse, it is thought that common lymphoid progenitors (CLP) emigrate from the bone marrow and enter the thymus. Upon colonizing the thymus, maturation of the CLP to mature CD4+ or CD8+ thymocytes is marked by sequential acquisition and loss of cell surface markers. The most immature thymocytes are negative for CD4 and CD8 and are termed double negative, or DN cells. The cells then acquire CD4 and CD8 and become double positive (DP) cells, and finally lose either CD4 or CD8, becoming CD8 or CD4 single positive (SP) cells respectively^5^. A series of studies has shown that a small number of thymic resident cells retain the potential to differentiate to alternative hematopoietic fates, including NK, B-cell, and myeloid lineages^6,7^.

As a first approximation, many cancers can be regarded as caricatures of normal differentiation, with the cancer cells now “frozen” at an immature stage of tissue-specific differentiation. Thus, understanding the biology and origin of cancer stem cells (CSCs) is an important step in elucidating the biology of cancer as well as developing new cancer therapies^8^. It has been proposed that most CSC originate from mutations that are acquired in stem cells that have an intrinsic property of self-renewal^9^. Alternatively, a multipotent committed progenitor cell, that lacks intrinsic self-renewal, can acquire mutations that lead to self-renewal^10^.

Acute myeloid leukemia (AML) is characterized by the uncontrolled expansion of myeloid stem and/or progenitor cells population in the bone marrow (BM). It is generally accepted that AML originates from malignant transformation of a hematopoietic stem or progenitor cell residing in the BM^11^. Following malignant transformation, the leukemic cells disseminate and invade tissues throughout the organism, producing symptoms that lead to the diagnosis.

A common genetic feature of many cases of AML are gross chromosomal rearrangements, including chromosomal translocations. These translocations typically generate chimeric fusion genes, which encode oncogenic fusion proteins. We have previously characterized leukemias initiated by NUP98-HOXD13 (NHD13) and NUP98-PHF23 (NP23) oncoproteins using transgenic mouse models, with expression directed to the hematopoietic compartment by Vav1 regulatory elements^12,13^. The NHD13 fusion is seen in human patients with MDS and AML^14,15^ and NHD13 transgenic mice develop MDS, which transforms to AML in approximately 60% of the mice^16,17^. The NP23 fusion, which is seen in human patients with AML or T-ALL^18^, leads to a wide spectrum of leukemia in mice, including myeloid, erythroid, T and B cell leukemia^13,19^. In our current study we characterize a unique phenotype seen in NP23-NHD13 double transgenic mice, which demonstrates that AML can be initiated in the thymus, as opposed to the BM of these mice.

## Results

### Co-expression of NP23 and NHD13 transgenes in the hematopoietic compartment leads to an aggressive AML with complete penetrance

**Figure 1:**
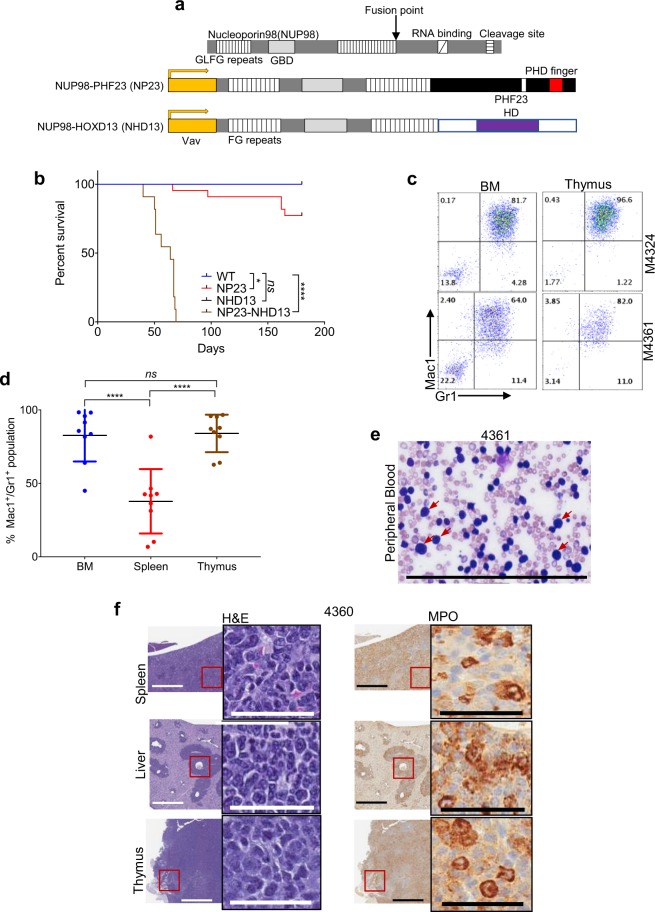
NP23-NHD13 mice develop aggressive AML. (**a**) Structure of NUP98 (top), NUP98-PHF23 fusion transgene (middle), and NUP98-HOXD13 fusion transgene (bottom). Protein fusion points are indicated by arrow; Vav regulatory elements are indicated in yellow. (**b**) Survival of NP23-NHD13(*n* = 11), NP23(*n* = 22), NHD13(*n* = 9) and WT(*n* = 17) mice. WT and NHD13 curves are superimposed at 100% survival each. Data analyzed by Log-rank (Mantel-Cox) test, ^****^P < 0.0001 (WT vs NP23-NHD13). (**c**) Thymus is more heavily invaded with Mac1+/Gr1 + leukemic cells than BM in several samples. (**d**) Tissue invasion of myeloid cells. Data are expressed as means ± SD, *n* = 9 mice. (**e**) Sheets of myeloblasts in peripheral blood smear; several examples indicated with red arrows. (**f**) H&E staining of leukemic cell invasion in parenchymal organs (spleen, liver and thymus); myeloperoxidase (MPO) staining of infiltrate. *ns* = not significant, *P < 0.05, ****P < 0.0001, by one-way ANOVA with a correction provided by the Tukey’s multiple comparisons test. Scale bars = 200 μm for blasts count, 800 μm (low power view), 50 μm (high power view).

Interbreeding the NP23 and NHD13 transgenic mice produced NP23-NHD13 double transgenic mice that expressed both NP23 and NHD13 fusion genes in hematopoietic tissues (Fig. 1a). NP23-NHD13 double transgenic mice showed markedly decreased survival (P < 0.0001) compared with their wild type (WT) and single transgenic (NP23, NHD13) littermates (Fig. 1b), suggesting a high penetrance of lethal disease. Median survival of NP23-NHD13 double transgenic progeny was 64 days, with some mice beginning to show signs of disease shortly after one month of age. Signs of disease included weight loss, lethargy, and hunched posture. Given the early fatalities in the initial litters of NP23-NHD13 mice, complete blood count (CBC) were monitored every two weeks after the first month of age for subsequent litters. NP23-NHD13 mice with disease showed a variable degree of anemia and thrombocytopenia and a consistent leukocytosis (Supplementary Table S1). Necropsy of diseased mice typically revealed hepatosplenomegaly or splenomegaly.

Unlike the single transgenic counterparts, which developed a wide, varied spectrum of different leukemias, the NP23-NHD13 mice uniformly developed acute myeloid leukemia (AML). The AMLs were characterized by a myeloblast (Mac1^+^/Gr1^+^) population that infiltrated the peripheral blood (PB), bone marrow (BM), spleen, thymus, and liver (Fig. 1c–f). It was not surprising that the Mac1^+^/Gr1^+^ population invaded PB, BM and spleen, a common phenomenon in murine AML^20^, and similar to AML driven by NP23 or NHD13 fusions. However, a prominent invasion of Mac1^+^/Gr1^+^ cells was observed in the thymus of double transgenic mice (Fig. 1c); in many cases, the invasion was more prominent in the thymus than BM (Fig. 1c and Supplementary Table S1), leading to the intriguing possibility that the AML in the NP23/NHD13 mice was derived from thymic-resident cells rather than BM-resident cells.

**Figure 2:**
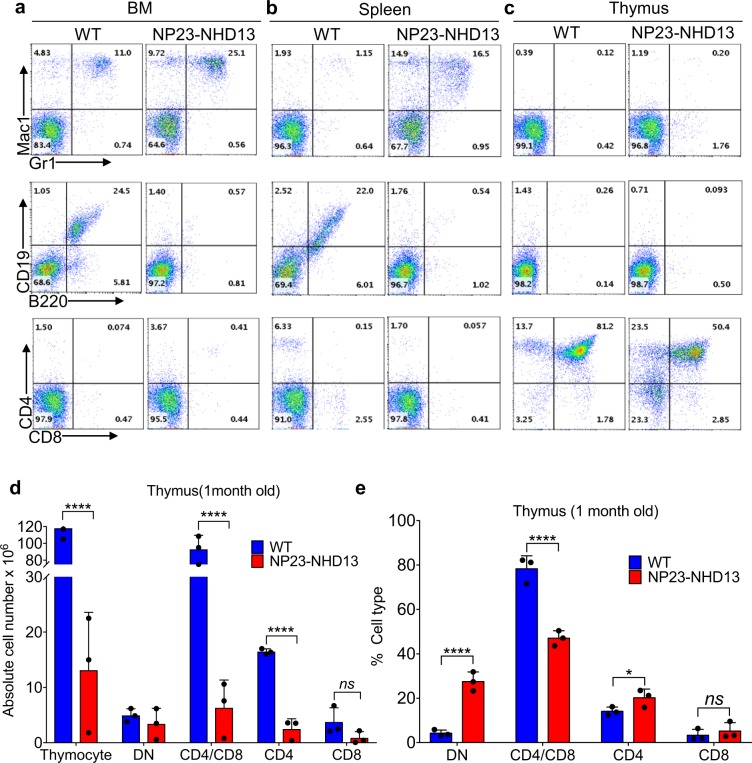
Non-leukemic, clinically healthy NP23-NHD13 mice are lymphopenic. (**a**–**c**) Flow cytometry of indicated tissues from young WT and NP23-NHD13 mice. (**d**) Absolute cell number of NP23-NHD13 thymus and thymic subsets (DN-double negative, CD4^+^, CD4^+^/CD8^+^ and CD8^+^) compared to WT. (**e**) Percentage of thymic subsets (DN-double negative, CD4^+^, CD4^+^/CD8^+^ and CD8^+^) compared to WT. Data are expressed as means ± SD, *n* = 3 mice per group. *ns* = not significant, *P < 0.05, ****P < 0.001, by one tailed student t-test.

We previously reported that both NP23^12^ and NHD13 mice^21^ showed a relative T-cell differentiation block at the DN2-DN3 transition; therefore, we analyzed thymic development of young (22–30 d), non-leukemic NP23-NHD13 mice. The young NP23-NHD13 mice showed normal hemoglobin and platelet counts but significantly reduced WBC, due primarily to markedly decreased circulating B and T-lymphocytes (Supplementary Table S2). Consistent with the CBC results, flow cytometry of BM, spleen, and thymus demonstrated a significant reduction of splenic B cells and thymic CD4^+^CD8^+^ DP thymocytes (Fig. 2a–c), as well as a relative increase in double negative (DN) thymocytes. There was no evidence of an increased number of Mac1^+^/Gr1^+^ myeloid cells in the thymus at this point in time (Fig. 2c). To better understand the marked decrease in both total thymocytes as well as mature CD4^+^ and CD8^+^ thymocytes (Fig. 2d–e), we fractionated the DN subpopulation from young NP23-NHD13 mice. Supplementary Fig. S1 shows an increase in DN2 but decrease in DN3 cells in the NP23-NHD13 thymus, suggesting a partial differentiation block at DN2-DN3 stage, with the resultant accumulation of DN2 cells.

To further characterize the AML thymic infiltrate, we searched for evidence of clonal Tcrb gene rearrangements in the thymic AML samples. Although we found no evidence of clonal Tcrb VDJ rearrangements in any sample, two thymic samples (#4352 and #4360) had clonal Tcrb D-J rearrangements (which begin at the DN2 stage of differentiation and invariably precede VDJ rearrangements (Supplementary Fig. S2A–C)^22^. However, Supplementary Fig. S3A,B show that although the BM from mouse #M4352 is almost completely replaced by Mac1^+^/Gr1^+^ leukemic cells, it is negative for a clonal Tcrb-DJ rearrangement, whereas the thymus, less infiltrated with Mac1^+^/Gr1^+^ AML is positive for a clonal Tcrb-DJ rearrangement. These findings suggested that the clonal DJ rearrangements in mouse #4352 were not derived from the Mac1^+^/Gr1^+^ leukemic clone, but rather from a distinct population of cells that were Mac1^−^/Gr1^−^ (Supplementary Figure S3A and S3B). To more clearly determine the origin of clonal Tcrb DJ rearrangements in the leukemic NP23-NHD13 mouse thymus, we euthanized two NP23/NHD13 mice with early AML (i.e, asymptomatic, but with increased WBC in peripheral blood), and sorted the thymic cells into Mac1^+^/Gr1^+^ and Mac1^−^/Gr1^−^ populations (Supplementary Fig. S3C). PCR followed by Sanger sequencing clearly showed that the clonal Tcrb DJ was present in the Mac1^−^/Gr1^−^ subset, but not the bulk Mac1^+^/Gr1^+^ AML clone (Supplementary Fig. S3D and S3E). The absence of this clonal marker in the bulk leukemic population strongly suggests that the Mac1^+^/Gr1^+^ AML did not evolve from a Mac1^−^/Gr1^−^ population with a clonal Tcrb DJ rearrangement but that the Mac1^−^/Gr1^−^ clone with Tcrb DJ rearrangement was rapidly out-competed by a genetically distinct Mac1^+^/Gr1^+^ AML clone.

### Gene expression signatures indicate that NP23-NHD13 AML is distinct from AML in NP23 or NHD13 single transgenic mice

**Figure 3:**
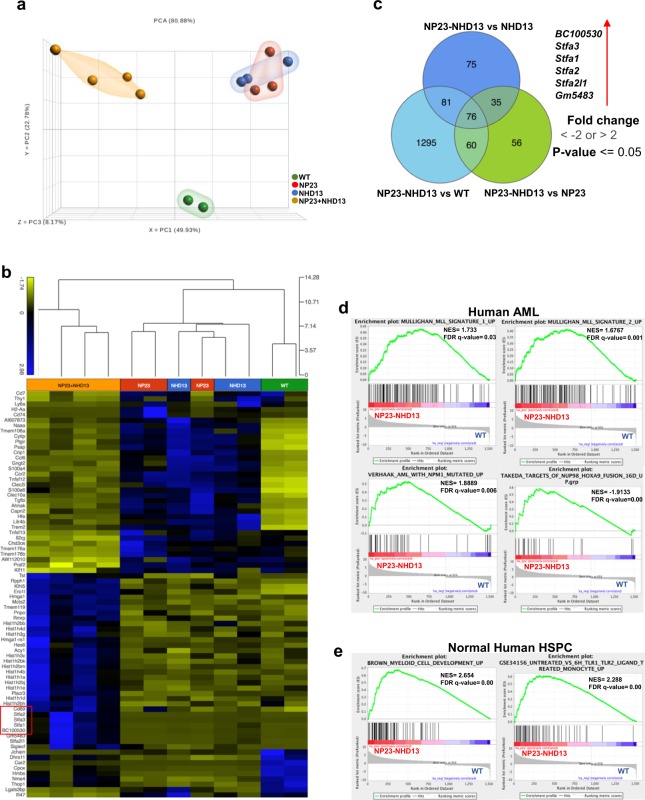
NP23-NHD13 AML gene expression profile is distinct from single transgenic NP23 and NHD13 AML. (**a**) PCA plot derived from NP23, NHD13 and NP23-NHD13 AML (all BM samples) compared to WT Lin^−^ BM. (**b**) Hierarchical clustering shows NP23-NHD13 leukemia samples cluster distinct from WT, NP23 and NHD13 leukemia samples. (**c)** Venn diagram indicates 76 genes differentially expressed in NP23-NHD13 AML. (**d**) GSEA comparison of genes differentially (defined as 2-fold expression change, p < 0.05) expressed between NP23-NHD13 AML and WT LN BM demonstrates that gene sets upregulated in NP23-NHD13 AML are also enriched in specific human AML gene sets, (**e**) Enrichment of genes overexpressed in NP23-NHD13 AML correlate with genes enriched in normal human myeloid development.

To further characterize the aggressive AML that arose in the NP23-NHD13 thymus, the gene expression profile was assessed in bulk AML cells arising from NP23-NHD13 (n = 4), NHD13 (n = 3) and NP23 (n = 3) mice and compared to WT lineage negative (LN) BMNC (n = 2; a third sample failed quality control and was excluded). Although we suspect that the NP23-NHD13 AML originated in the thymus, we used AML samples harvested from the BM to ensure consistent background elements. Principle Component Analysis (PCA) shows that the NP23-NHD13 AML samples clustered together and away from NP23 and NHD13 AML samples (which were intermingled), suggesting that the AML which developed in NP23-NHD13 mice was different than the AML that developed in NP23 and NHD13 mice (Fig. 3a). A heat map showing differential gene expression is shown in Fig. 3b, and a Venn diagram identified 76 genes that were differentially expressed in NP23-NHD13 AML compared to the other three groups (Fig. 3c and Supplementary Table S3). Among the differentially expressed genes, those encoding StefinA proteins (*Stfa1*, *Stfa2*, *Stfa3*, *BC100530*, *Gm5483 and Stfa2l1*) were consistently overexpressed in NP23-NHD13 mice, up to 1000-fold higher than the WT LN BMNC (Fig. 3b; Supplementary Table S3). Of note, CD69, a gene whose expression is normally restricted to developing thymocytes and T cells, was uniquely overexpressed in the NP23-NHD13 AML samples compared to WT LN BMNC as well as NP23 or NHD13 AML. Gene set enrichment analysis (GSEA) showed the NP23-NHD13 AML expression profile to be enriched in human AML, specifically including subsets of MLL-fusion AML, NPM1-mutant AML, and NUP98-HOXA9 fusion AML (Fig. 3d). In addition, the NP23-NHD13 AML showed upregulation of genes involved in normal myeloid development (Fig. 3e).

We used nanostring technology as an orthogonal approach to compare gene expression in NP23-NHD13 AML (n = 3) to WT lineage negative (LN) BMNC (n = 3), using a custom set of 109 genes involved in hematopoietic differentiation (Supplementary Table S4). Several genes known to be important for myeloid differentiation, including Pu.1, Id1, Cebpa, Csf2rb, Csf1r, Gfi1, Itgam, and Hoxa cluster genes were at least 2-fold upregulated in the NP23-NHD13 AML, using both assays (Supplementary Fig. S4).

### Whole Exome sequencing (WES) did not identify any additional collaborative mutations in NP23-NHD13 double transgenic mice

We suspected that acquisition of spontaneous, collaborative mutations may be required for malignant transformation of normal T-cell precursors to AML and used whole exome sequencing (WES) to identify mutations in the BM of 16 primary NP23-NHD13 leukemic mice. Tier 1 (coding) SNV were identified and compared to C57BL6-WT reference database. Although we found rare mutations in known cancer genes, such as Cbl, Mybl2, and Stat1, we found no recurrent tier 1 mutations amongst the 16 samples, suggesting that additional acquired mutations may not be required to release the leukemic potential of NP23-NHD13 hematopoietic cells, and that concurrent expression of NP23 and NHD13 in the mouse thymus may be sufficient for trans-differentiation and malignant transformation (Supplementary Table S5).

### NP23-NHD13 AML cells are transplantable

**Figure 4:**
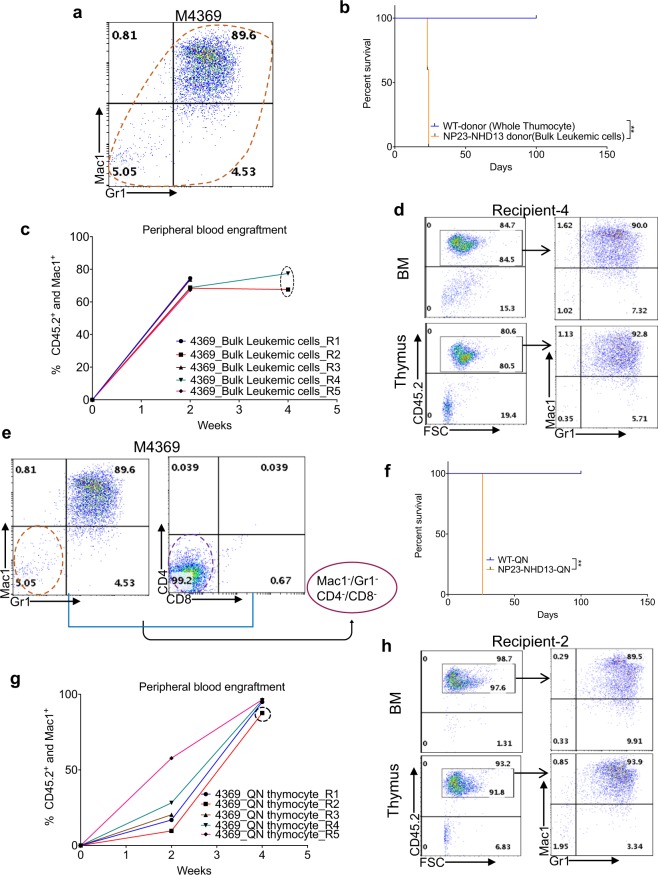
Thymic AML is transplantable. (**a**) Flow cytometry of thymus invaded by AML cells (#4369). (**b**) Survival of WT (total thymocytes) and NP23-NHD13 (bulk thymic AML cells) transplant recipients, 1 × 10^6^ cells /mouse, *n* = 5 mice per group. Data are analyzed by Log-rank (Mantel-Cox) test. (**c**) Engraftment of CD45.2^+^/Mac1^+^ cells at indicated time points (dotted circle at 4 wk time point indicates engraftment data from BM). (**d**) Flow cytometry of representative transplant recipient (R-4) shows engraftment of leukemic myeloid cells in both BM and thymus. (**e**) Quadruple negative (QN) thymocytes (Mac1^−^/Gr1^−^ and CD4^−^/CD8^−^, 8.4 × 10^4^ cells/mouse) transplanted from donor #4369, WT-QN thymocytes (9.6 × 10^4^ cells/mouse) are used as control, *n* = 5 mice per group. (**f**) Survival of WT and NP23-NHD13 (QN) transplant recipients analyzed by Log-rank (Mantel-Cox) test. (**g**) Engraftment of CD45.2^+^/Mac1^+^ cells at indicated time points (dotted circle at 4 wk time point indicates engraftment data from BM). (**h**) Flow cytometry of representative transplant recipient (R-2) indicates engraftment and myeloid commitment of the transplanted QN thymocytes in BM and thymus. **P < 0.01.

Given that transplantation of AML to WT recipients has been suggested to be a cardinal feature of AML in mice^20^ unselected AML cells from the thymus of a NP23-NHD13 leukemic mouse (#4369; Fig. 4a) were transplanted into WT recipients following sublethal irradiation with 600 cGy. The donor mice express the CD45.2 allele of CD45, whereas the recipient mice express CD45.1(Supplementary Fig. S5), allowing discrimination of donor and recipient hematopoiesis with CD45.2 and CD45.1 antibodies. As seen in Fig. 4b,c, all mice engrafted rapidly and died within 4 weeks of an invasive AML (Fig. 4d), with replacement of normal BM and thymus with Mac1^+^/Gr1^+^ AML.

To investigate the possibility that AML in the NP23-NHD13 mice might originate from thymic progenitors, we transplanted quadruple negative (QN) thymocytes (Mac1^−^/Gr1^−^ and CD4^−^/CD8^−^) from the same leukemic mouse (Fig. 4e). Survival (Fig. 4f) and engraftment were almost identical to the bulk leukemic cells, except for lower engraftment at 2 weeks (Fig. 4g). One animal was found dead at 26 days, and the remainder were euthanized as CBCs showed findings consistent with AML (Supplementary Table S6). Flow cytometry revealed invasion of BM and thymus with Mac1^+^/Gr1^+^ cells (Fig. 4h), leading to the conclusion that the Mac1^+^/Gr1^+^ AML originated from thymic resident QN cells. To determine if the transplant recipients showed a gene expression profile similar to the primary leukemic mice, RNASeq was performed with AML samples collected from BM of leukemic QN recipients (R1, R2, R4, n = 3). Hierarchical clustering demonstrated that the gene expression profile from transplant recipients and primary NP23-NHD13 AML clustered together (Supplementary Fig. S6). Taken together these results indicate that both bulk AML cells as well as QN thymocytes were able to transmit AML; of note, 10-fold fewer QN thymocytes were able to transmit AML with a time course identical to that of the bulk AML cells.

### Thymic cells from a non-leukemic NP23-NHD13 mouse also produced AML in transplant recipients

**Figure 5:**
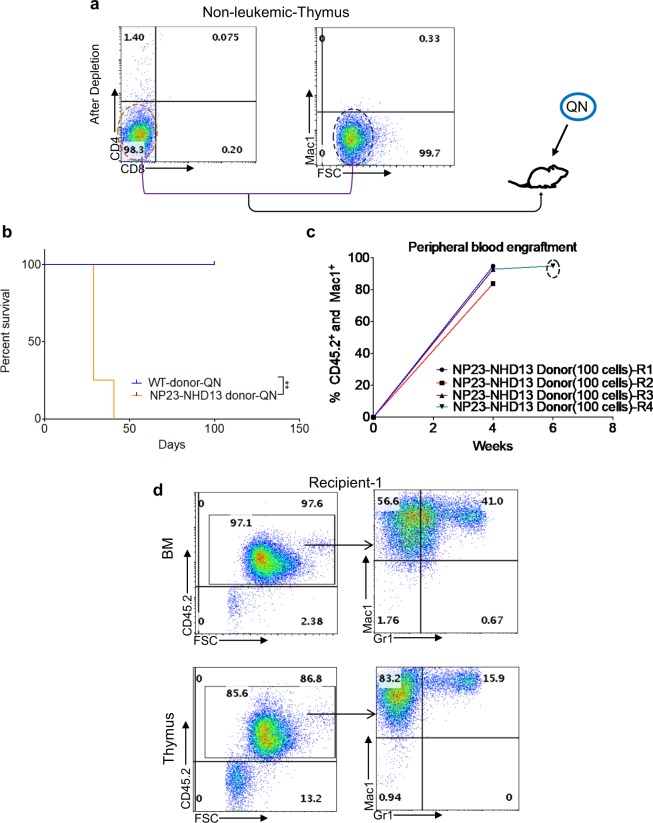
Transplantation of QN thymocytes from non-leukemic NP23-NHD13 thymic cells leads to AML. (**a**) Purification of QN thymocytes from non-leukemic thymus and transplantation of 100 cells per recipient. (**b**) Survival curve of WT and NP23-NHD13 QN recipients, *n* = 4–5 mice per group. Data are analyzed by Log-rank (Mantel-Cox) test. **c** Engraftment of Ly45.2^+^/Mac1^+^ cells at indicated time interval (dotted circle indicates engraftment data obtained from BM). (**d**) Flow cytometry profile of representative transplant recipient (R-1) indicates engraftment and differentiation of QN thymocytes in both BM and thymus. ^**^P < 0.01.

To verify that the AML produced by the QN thymocyes from #4369 were not caused by contaminating Mac1^+^/Gr1^+^ AML cells, we transplanted QN thymocytes (100 cells/mouse) from a one-month old NP23-NHD13 mouse with no signs of AML (Fig. 5a). All recipients developed AML within 6 weeks (Fig. 5b,c), characterized by anemia, thrombocytopenia, leukocytosis (Supplementary Table S7), and infiltration of BM and thymus with myeloid cells (Fig. 5d). These results demonstrate that non-leukemic QN thymocytes could also generate AML in a cell-autonomous fashion.

### Both Mac1^+^/Gr1^+^ and Mac1^−^/Gr1^−^ populations produce AML in recipients

The above experiments demonstrate the leukemic potential of the QN thymic cells but did not evaluate the leukemic potential of the more mature Mac1^+^/Gr1^+^ population, leaving open the possibility that the bulk Mac1^+^/Gr1^+^ cells were committed, non-self-renewing cells unable to propagate the leukemia. Therefore, we purified and transplanted the Mac1^−^/Gr1^−^ and Mac1^+^/Gr1^+^ fraction from the thymus (Supplementary Fig. S7a) of a leukemic NP23-NHD13 mouse. The survival curve shows that both fractions transmitted AML in recipients although the Mac1^−^/Gr1^−^ fraction showed a modestly increased latency (Supplementary Fig. S7b). Flow cytometry of BM and thymus showed that both the Mac1^+^/Gr1^+^ and Mac1^−^/Gr1^−^ fraction produced donor derived AML (Supplementary Fig. S7c).

### DN thymic progenitor cells from NP23-NHD13 mice retain abundant myeloid differentiation potential *in vitro*

**Figure 6:**
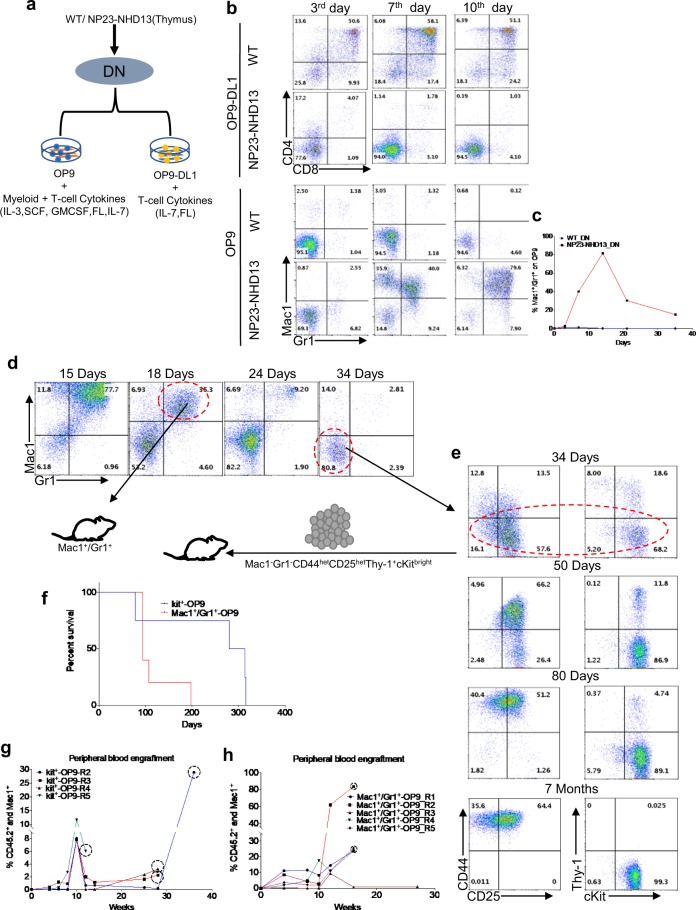
*In vitro* differentiation of NP23-NHD13 DN thymocytes. **(a**) *In vitro* co-culture of DN cells from WT and NP23-NHD13 mice on OP9 and OP9-DL1 feeder layers with the indicated cytokines; DN cells were also Mac1^−^(1 × 10^5^ cells were plated). (**b**) Differentiation of DN cells on OP9 and OP9-DL1 at indicated timepoints. (**c)** graph represents Mac1+/Gr1 + percentage of WT and NP23-NHD13 DN cells on OP9. (**d**,**e**) Long term co-culture of DN cells from NP23-NHD13 mice on OP9. Mac1^+^/Gr1^+^ and Mac1^−^Gr1^−^CD44^het^CD25^het^Thy-1^+^cKit^bright^ (designated Kit + -OP9) cells (1 × 10^4^ cells/mouse) were transplanted at 18 or 34 days of culture, respectively. (**f**) Survival of Mac1^+^/Gr1^+^ and Kit + -OP9 recipients. Data are analyzed by Log-rank (Mantel-Cox) test), *P* = 0.07. Engraftment of donor myeloid cells (CD45.2^+/^Mac1^+^) is shown in both (**g**) Kit + -OP9 and (**h**) Mac1^+^/Gr1^+^ recipients (dotted circle indicates engraftment data obtained from BM).

Murine thymocyte differentiation is marked by an orderly acquisition and loss of cell surface markers^23^ (Supplementary Fig. S8), and irreversible commitment to the T-cell lineage is thought to occur at the DN2-DN3 transition^24^. The observation that cells from the thymus could generate AML in a cell-autonomous manner was surprising, but not completely unprecedented. OP9 feeder cells have been used to reveal the myeloid potential of WT thymocytes^25^, whereas OP9-DL1 cells, which stimulate Notch1 pathway activation, support normal thymocyte maturation to T-cells^26^ (Fig. 6a). To determine the differentiation potential of DN thymocytes from NP23-NHD13 mice with and without enforced Notch1 signaling, purified DN cells from WT and NP23-NHD13 thymus (1 × 10^5^/plate) were cocultured on OP9 and OP9-DL1 feeder layers, with myeloid or T-cell supportive cytokines respectively (Fig. 6a). WT DN cells plated on OP9-DL1 differentiated into DP thymocytes whereas NP23-NHD13 DN cells showed no evidence of differentiation to DP thymocytes (Fig. 6b). Conversely, although WT thymocytes showed rare myeloid differentiation, consistent with previous reports^25^ the NP23-NHD13 DN cells produced abundant Mac1^+^/Gr1^+^ cells (Fig. 6b,c).

To determine if the marked expansion of Mac1^+^/Gr1^+^ cells was persistent, we again co-cultured DN cells from NP23-NHD13 and WT thymi on an OP9 feeder layer. The NP23-NHD13 DN thymocytes produced Mac1^+^/Gr1^+^ cells for at least 24 days (Fig. 6d). However, by 34 days, production of Mac1^+^/Gr1^+^ cells were markedly diminished, although the co-cultured cells continued to expand. The immunophenotype of the expanding cells did not consistently match that of any normal developing thymocyte subset, as Thy1, CD44, and CD25 expression varied with time. However, these cells were consistently Kit positive; therefore, we refer to these Mac1^−^/Gr1^−^/Kit^+^ as Kit^+^-OP9 cells (Fig. 6e).

To investigate the leukemic potential of both the Mac1^+^/Gr1^+^ cells that predominated during the initial 18 days of NP23/NHD13 DN thymocyte co-culture, as well as the Kit^+^-OP9 cells that predominated after day 34, we transplanted cells from day 18 and day 34 OP-9 co-cultures (Fig. 6d,e). Both Mac1^+^/Gr1^+^ (day 18) and Mac1^−^/Gr1^−^/Kit^+^/(day 34) populations transmitted disease in recipients although the median survival (297d) of the Kit^+^-OP9 (day 34) population was markedly longer than the median survival of the Mac1^+^/Gr1^+^ population (94d) (Fig. 6f), and the survival of both groups was markedly longer than recipients of primary NP23-NHD13 thymocytes (26–42 d; Figs. 4 and 5). Peripheral blood showed engraftment of donor-derived myeloid cells in recipients of both Mac1^+^/Gr1^+^ cells and Kit^+^-OP9 cells (Fig. 6g–h). Interestingly, eight of nine recipients developed slowly progressive pancytopenia, BM cellularity, blast percentage (mean 92, range (80%-98%), and histology that were consistent with AML evolved from MDS (Supplementary Table S8 and Supplementary Fig. S9a–i); leading to the speculation that the NP23-NHD13 cells had lost the ability to rapidly and aggressively expand during the *in vitro* culture period.

### The thymic resident AML leukemia initiating cell (LIC) resides in the DN1 and DN2 populations

**Figure 7:**
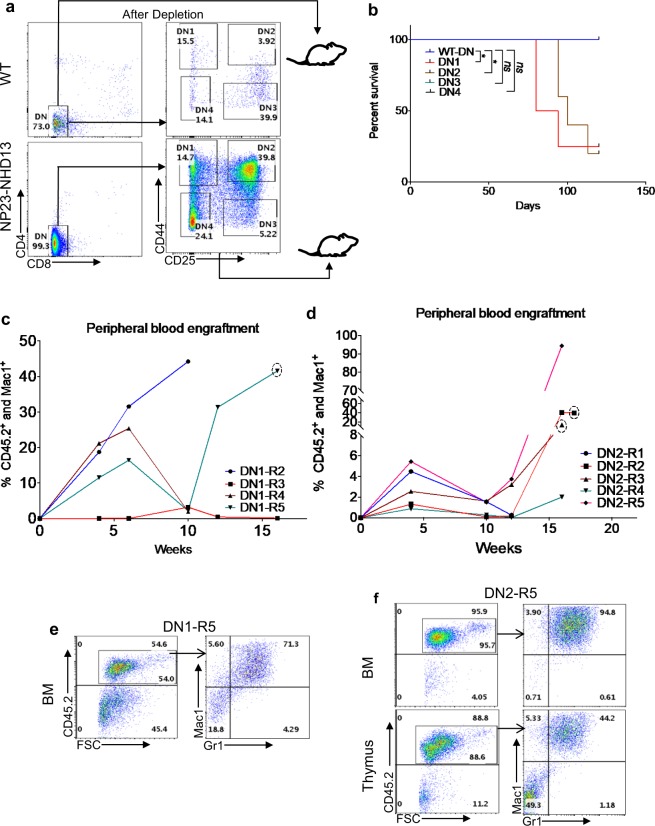
DN1 and DN2 thymocytes from NP23-NHD13 mice transmit AML. (**a**) Sorting strategy for purification of DN populations. The entire sorted population was transplanted (DN1 = 8.7 × 10^3^/recipient, DN2 = 3.7 × 10^4^/recipient, DN3 = 5 × 10^3^/recipient, DN4 = 2.4 × 10^4^/recipient, WT-DN = 10^5^cells/recipient), *n* = 4–5 mice per group. (**b**) Survival curve shows DN1 and DN2 recipients develop disease. Data analyzed by Log-rank (Mantel-Cox) test. (**c**,**d**) Biphasic engraftment pattern of donor cells (CD45.2) is shown in both DN1 and DN2 recipients, (dotted circle indicates engraftment data obtained from BM). (**e**) Flow cytometry of transplant recipient mice from DN1 and (**f**) DN2 recipients demonstrate engraftment of donor derived AML cells. *P < 0.05.

We hypothesized that progenitor thymocytes, but not committed thymic progenitors would generate AML. We transplanted purified DN1(CD44^+^CD25^−^), DN2(CD44^+^CD25^+^), DN3(CD44^−^CD25^+^), and DN4(CD44^−^CD25^−^) populations from NP23/NHD13 mice, using WT DN cells as a control (Fig. 7a). Only the DN1 and DN2 thymocytes produced lethal disease in recipients (Fig. 7b); the DN3 and DN4 recipients showed no evidence of engraftment at any time point (data not shown). Interestingly, serial engraftment assays revealed that many recipients seemed to show two “waves” of engraftment (Fig. 7c,d), perhaps reflecting variable self-renewal potential of the transplanted thymocytes. DN1 and DN2 recipients showed elevated WBC, decreased hemoglobin, and decreased platelets (Supplementary Table S9). BM and thymus from DN1 and DN2 recipients were invaded with AML cells of donor origin (Fig. 7e,f); histology and immunohistochemistry also confirmed infiltration of myeloid cells (Supplementary Figs. S10a and S10b).

DN1 cells have been divided into sub-populations designated DN1a (CD44^+^CD25^−^cKit^high^CD24^−^), DN1b(CD44^+^CD25^−^cKit^high^CD24^lo^), DN1c(CD44^+^CD25^−^cKit^lo^CD24^+^), DN1d(CD44^+^CD25^−^cKit^−^CD24^+^) and DN1e(CD44^+^CD25^−^cKit^−^CD24^−^) thymocytes^27^. Young (one month old), non-leukemic NP23-NHD13 mice showed an increase in the DN1a and b and decrease in the DN1d populations (Supplementary Fig. S11a,b). The DN1 subpopulations were sorted and transplanted; only the DN1a and DN1b subpopulations (both Kit high) transmitted disease (Supplementary Fig. S11c), and the engraftment assay again showed a suggestion of two waves of engraftment (Supplementary Fig. S11d and S11e). In addition to engraftment of CD45.2^+^ cells in the peripheral blood, CBC showed abnormal Hgb and neutrophil count in the DN1a and DN1b recipients, while the DN1c and DN1d recipients showed no engraftment and normal CBCs (Supplementary Table S10). At time of death, flow cytometry of DN1a and DN1b recipients showed tissue invasion of donor-derived CD45.2^+^ Mac1^+^/Gr1^+^ AML cells (Supplementary Figs. S10f and S11g) as well as elevated WBC, decreased hemoglobin, and decreased platelets (Supplementary Table S10). Taken together, these results demonstrate that progenitor thymocytes (DN1a, DN1b, and DN2) but not committed thymic progenitor (DN3, DN4) from NP23-NHD13 mice could generate AML in a cell-autonomous manner.

### Cells resembling MPP2/3 cells are abundant in the NP23-NHD13 thymus

**Figure 8:**
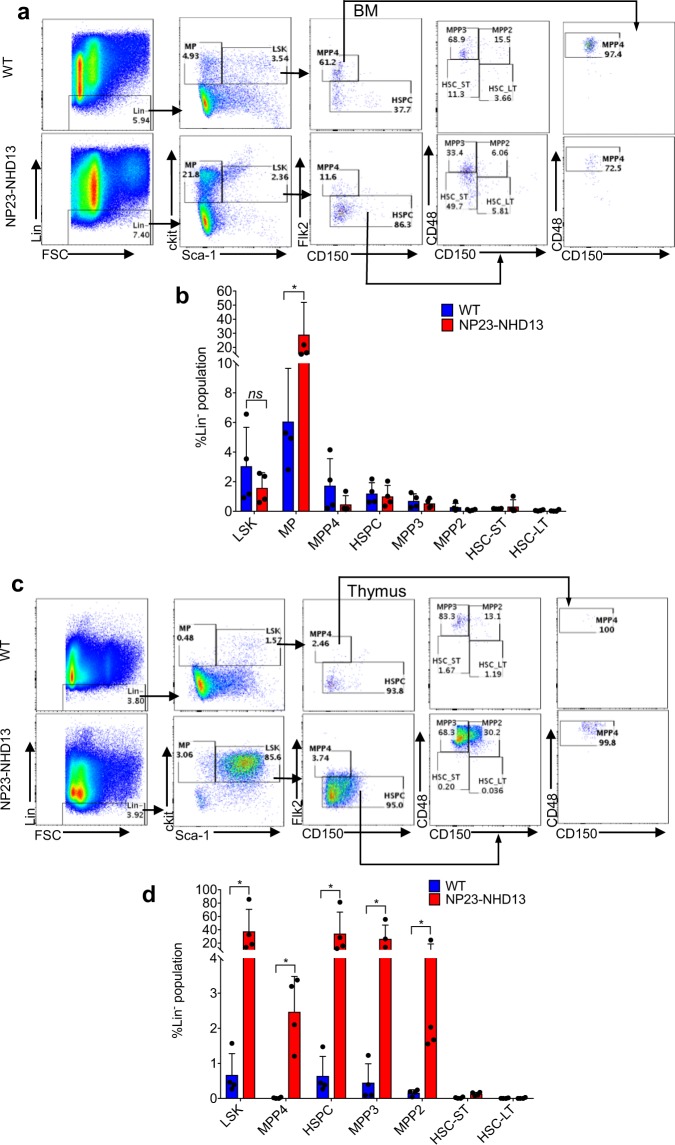
Thymi from non-leukemic NP23-NHD13 mice show a markedly expanded LSK compartment. (**a**) Flow cytometry of WT and NP23-NHD13 BM using Flk2, CD48, and CD150 staining to fractionate LSK cells (**b**) Quantification of LSK cells and LSK subpopulations in BM ^ns^*P* = 0.17(LSK). (**c)** Flow cytometry of WT and NP23-NHD13 thymus (**d**) Quantification of LSK cells and LSK subpopulations in thymus. Data are expressed as means ± SD, *n* = 4 mice per group, *P < 0.05, by Mann Whitney test.

To better characterize the LIC present in the thymus of NP23-NHD13 mice, we evaluated the thymocytes from NP23-NHD13 mice using antibody staining strategies typically employed to characterize murine HSPCs^28^. We first characterized WT and NP23-NHD13 BM using Flk2, CD48, and CD150 surface staining to verify that our staining protocol was consistent with published results. Compared to WT BM, NP23-NHD13 mice showed a marked increase in Lin-Kit + Sca- myeloid progenitor (MP) cells, and similar numbers of Lin-Sca + Kit + (LSK) cells and LSK subsets, including MPP2, MPP3, MPP4, ST-HSC, and LT-HSC (Fig. 8a,b). However, the NP23-NHD13 thymus showed a marked (45 fold) expansion in the proportion of LSK cells in the thymus (Fig. 8c,d). Fractionation of the thymic LSK compartment showed that the dramatic increase in the LSK population was principally due to an expansion of cells with the staining characteristics of MPP2 and MPP3 cells (Fig. 8c). To further characterize this unusual thymic LSK population we assessed expression of CD25, a subunit of interleukin 2 (IL-2R) receptor normally expressed on thymic progenitor cells (DN2 and DN3)^29^. The BM resident HSPC from both WT and NP23-NHD13 mice did not express CD25 (Supplementary Fig. S12a). However, the markedly expanded MPP2 and MPP3 populations in the NP23-NHD13 thymus were almost entirely CD25+ (Supplementary Figs. S12b and S12c).

### Thymic resident LSK subpopulations engraft and produce AML

To determine if the thymic resident LSK populations could engraft and differentiate along the myeloid lineage, we transplanted flow sorted LSK cells from BM and thymus of a young (1month old) NP23-NHD13 mouse; WT BM LSK cells were used as a control (Supplementary Fig. S13a). All transplant recipients showed engraftment at 4- and 8-weeks post-transplant (Supplementary Fig. S13b). One recipient from each donor source was euthanized to assess engraftment of BM and thymus. Supplementary Fig. S13c shows that NP23-NHD13 BM LSK and thymus LSK transplant recipients engrafted in the BM, but not the thymus. Although the most prominent population generated from the thymic and BM -derived LSK cells were Mac1+/Gr1+, approximately 10% of cells were CD71+/Ter119+ erythroid precursors. At 10 wks post-transplant, CBC from the BM and thymic LSK recipients revealed anemia and thrombocytopenia, which correlated with the degree of engraftment evident in the peripheral blood (Supplementary Table S11).

Two recipients of either BM or thymic LSK cells from NP23-NHD13 donors developed lethargy and/or progressive anemia and were euthanized; the remaining mice were alive and well 16 weeks post-transplant. Flow cytometry of BM from both the thymic and BM LSK recipients were similar, and showed 11–24% engraftment of donor cells, and the presence of both Mac1+ myeloid cells and CD71+/Ter119+ erythroid cells (Supplementary Fig. S14a). CBC and BM morphology showed anemia, leukocytosis, and/or increased blasts in the BM, consistent with acute leukemia (Supplementary Fig. S14b,c). However, flow cytometry of thymus from the thymic LSK recipients was heavily engrafted (89–90% Ly5.2+), whereas the thymus from the BM LSK recipients was very poorly engrafted (0–3% Ly5.2+). Flow cytometry demonstrated that the engrafted thymic cells were primarily LSK cells, with the staining characteristics of MPP3/MPP2 cells (Supplementary Fig. S15). Taken together, these findings indicate that the thymic LSK cells colonize both the thymus and BM, and that the thymic cells which colonize and expand in the thymus are immunophenotypically similar to the LSK cells initially transplanted.

### Limiting dilution indicates that LIC are more frequent in thymus than BM

Although the above experiments demonstrate the presence of LIC in the thymus, we also detected LIC in the BM. To determine the relative frequency of LIC in BM and thymus, we euthanized a NP23-NHD13 mouse with AML (Supplementary Fig. S16a), and injected recipients with a limiting dilution series of BMNC or thymocytes. Engraftment was defined as >0.3% CD45.2 cells in the peripheral blood. The estimated LIC frequency in the thymus was 1/32 cells (range 1/10.2–1/103), similar to prior experiments in which we transplanted leukemic cells from the thymus (Fig. 4, Supp Fig. S7), whereas the estimated frequency in BMNC was 1/441 cells (range 1/123-1/1585) (Supplementary Fig. S16b), indicating that the LIC were more frequent in thymus than BM. Frequency calculation was performed using Walter + Eliza Hall Bioinformatics ELDA: Extreme Limiting Dilution Analysis Tool (http://bioinf.wehi.edu.au/software/elda/).

## Discussion

AML has historically been interpreted as a disease in which a hematopoietic stem or progenitor cell (such as an HSC, or CMP/GMP) undergoes malignant transformation in the bone marrow microenvironment, expands within the bone marrow, leading to reduction in physiologic hematopoiesis, and subsequently metastasizes systemically^30,31^. In this study, we show that mice which express both NP23 and NHD13 transgenes uniformly develop a highly aggressive AML, and that this AML originates from cells located within the thymus. Of note, the bulk leukemic population had features typical of AML, with blast morphology, Mac1 and Gr1 co-expression, myeloperoxidase positivity, invasion of parenchymal organs, and the ability to transmit leukemia to WT recipients. However, the cells that initiate the AML do not express myeloid cell surface antigens, but instead resemble LSK cells, most specifically a subset similar to MPP2 or MPP3 cells. In contrast to BM-resident LSK cells, the thymic resident LSK cells express high levels of the IL2 receptor protein CD25. In addition, although both BM cells and thymic cells could initiate AML, limiting dilution assays demonstrated that the leukemia initiating cells (LIC) were 14-fold more frequent in the thymus than the BM.

Gene expression profile of the AML that develops in the NP23-NHD13 mice closely matched gene expression profiles of several human AML subsets, including those with NPM1 mutations, MLL fusions, and NUP98 fusions. Additionally, there was strong enrichment for genesets upregulated during normal human myeloid development. Most striking was the dramatic elevation of stefin genes (Stfa1,2,3, and 2L1). These genes were upregulated 1000-fold compared to WT LN BM, and up to 250-fold compared to NHD13 or NP23 single transgenic AML. The stefin proteins are cysteine protease inhibitors that belong to the cystatin family of protease inhibitors^32^. Of the stefin family proteins, Stfa2L1 has been noted to be exclusively expressed in neutrophils amongst all hematopoietic subsets studied by the Immunological Genome (ImmGen) Project^33^. Consistent with previous results that indicated PU.1 (Spi1, Sfpi1) expression was a critical feature in myeloid differentiation from immature thymocytes^34^ PU.1 was one of the most significant differentially expressed genes in both the RNA-seq data as well as the Nanostring data. Notably absent from the genes upregulated in the NP23-NHD13 AML were genes involved in specification of normal immature thymocytes, such as Runx1, Ets1, Tcf3, Tcf7, Tcf12, Gata3, and Bcl11b^35^. The absence of a residual thymocyte gene expression signature stands in contrast to the retention of a thymocyte signature following thymocyte trans-differentiation to the myeloid lineage triggered by the co-expression of Myc and Bcl2 in purified DN2 thymocytes^36^.

There were very few acquired mutations in the NP23-NHD13 AMLs; of the 16 primary AML samples analyzed, we found only a single mutation of a gene commonly involved in human or murine AML (Cbl). This lack of acquired mutations contrasts with our prior WES studies in murine leukemias initiated by NUP98 fusions genes, in which we found spontaneous, acquired mutations of well-known leukemia genes (such as Nras, Kras, Idh1, Jak1/2, and Bcor) in 50% of AML^19^, 72% of ETP-ALL (L. Goldberg and P.D. Aplan, unpublished), and 100% of B cell precursor ALL^19^. These findings suggest that the combination of NP23 and NHD13 transgenes are sufficient to cause AML with 100% penetrance, and additional, spontaneous, somatic mutations are not required for malignant transformation. In retrospect, this may not be surprising, given the complete penetrance and early age (<3 months) of disease onset.

The lack of Tcrb gene rearrangements in the primary AML samples, is consistent with the observation that DN1 and DN2 cells transmitted AML to WT recipients, while DN3 and DN4 did not. These findings both indicate that the cell which transforms to AML is a thymic resident cell that has not differentiated beyond the DN2 stage.

Although rare, there are numerous case reports of AML involving the thymus or mediastinum without evidence of BM involvement, suggesting that the leukemia may have been initiated in the thymus of these patients^37–40^. These leukemias have been referred to as myeloid sarcoma or mediastinal granulocytic sarcoma. More common are cases of AML with both thymic or mediastinal involvement and BM infiltration^41^, most notably patients with *PICALM-MLLT10* (*CALM-AF10*) fusions. Patients with *PICALM-MLLT10* fusions typically have a mediastinal mass evident at the time of diagnosis, with T cell receptor gene rearrangements and expression of T cell antigens, and a diagnosis of T-cell ALL^42,43^. However, a subset of patients with a *PICALM-MLLT10* fusion (3 of 23 in one series) have a mediastinal mass, no expression of T-cell antigens, and positive expression of myeloid antigens such as CD33, CD34, or MPO, and were given a diagnosis of M1 AML or acute undifferentiated leukemia (AUL)^43^. Interestingly, patients with *PICALM-MLLT10* fusions overexpress *HOXA* cluster genes including *HOXA7*, 9, 10^44,45^ similar to the NP23-NHD13 mice characterized in this report, suggesting importance of *HOXA* cluster gene overexpression in the genesis of a thymic AML.

We have characterized a form of highly aggressive AML that appears as typical AML based on morphology, immunophenotyping, and immunohistochemistry, that originates in the thymus. Further characterization indicates that this form of AML is initiated by an undifferentiated thymocyte, that then partially differentiates (acquires myeloid antigens and a myeloid gene expression profile) along the myeloid lineage but retains the ability to self-renew and initiate AML. These findings, along with the clinical reports described above, suggest that the thymus should be regarded as a potential reservoir of AML cells in those patients who present with a mediastinal mass.

## Methods

### Generation of NP23-NHD13 double transgenic mice

The NP23 and NHD13 single transgenic mice were previously described^13,16^ and have been maintained in NIH animal facilities. NP23-NHD13 double transgenic mice were generated by breeding mice with a single NHD13 allele to mice with a single NP23 allele. The double transgene did not appear to cause embryonic lethality, as the mice were born at Mendellian ratios and were healthy for the first month of life. Genotyping primers are listed (Supplementary Table S12). Animal studies were approved by the National Cancer Institute (NCI) Intramural Animal Care and Use Committee, and all experiments were performed in accordance with the relevant guidelines and regulations.

### Leukemia evaluation

Mouse health was monitored by careful observation and blood sample analysis by complete blood counts (CBCs). Mice that displayed non-specific signs of illness such as weight loss, lethargy, kyphosis or were moribund, were euthanized immediately. Upon necropsy, clinical signs consistent with leukemia, such as hepatosplenomegaly, lymphadenopathy, or thymic enlargement were noted. Tissues harvested were used for diagnostic assays including flow cytometry, histology, immunohistochemistry and were stored for DNA, RNA, and protein analysis. Diagnosis of AML was based on the “Bethesda Proposals^20^.

### Flow cytometry and cell sorting

Flow cytometry was performed as described^46^ with the following anti mouse conjugated antibodies (eBioscience or BD Biosciencies): Mac-1-phycoerytrin(PE)(Clone-M1/70), Mac-1-allophycocyanin(APC)(Clone-M1/70),Gr-1-fluorescein isothiocyanate (FITC)(Clone-RB6-8C5) CD4-PE(Clone-RM4-5), CD4-eFluor-450(Clone-RM4-5), CD8-PE(Clone-53-6.7), CD8-APC(Clone-53-6.7), CD8-APC-Cy7(Clone-53-6.7), Gr-1-APC-Cy7(Clone-RB6-8C5) CD19-PE (Clone-1D3), CD19-APC(Clone-MB19-1), B220-FITC(Clone-RA3-6B2),B220-APC-Cy7(Clone-RA3-6B2), CD71-PE(Clone-R17217), CD71-APC(Clone-R17217), Ter119-FITC(Clone-Ter-119), Ter119-APC(Clone-Ter-119) cKit-APC-Cy7(Clone-2B8) Sca-1-PE-Cynine7(PerCp-Cy7)(Clone-D7), CD16/32-PE(Clone-93), CD24-PE(Clone-30-F1) CD150-APC(Clone-TC15-12F12.2), Flk2-BV421(Clone-A2F10.1), CD48-FITC(Clone-HM48-1), CD34-FITC(Clone-RAM34), CD25-PE (Clone-PC61.5), CD25-APC-Cy7(Clone-PC61), CD44-FITC (Clone-IM7), CD44-APC(Clone-IM7), Streptavidin-APC(Cat#17-4317-82), Streptavidin-PE(Cat#12-431-87), Streptavidin-PerCp-Cyanine5.5(PerCp5.5). CD45.1-APC(A20), CD45.2-APC (Cat#1800-15). For sorting DN and DN1 subsets; DN thymocytes were enriched by staining with CD4 (L3T4), CD8a (Ly-2), and CD11b (Microglia) Microbeads (Miltenyi biotec), and depletion of the stained cells using MACS LD column (Miltenyi biotec), according to the manufacture’s protocol. The depleted cells were then stained with specific antibodies and sorted with an Aria Violet-Cli FACS sorter. BM subpopulation were sorted by harvesting BM from two femora and tibiae followed by staining with mouse lineage biotin antibody cocktail (Miltenyi biotec), according to manufacturer’s recommended protocol, along with other different lineage markers. Finally, the stained samples were sorted with an Aria Violet-Cli FACS sorter.

### Histology and immunohistochemistry

Tissues were fixed in 10% NBF and embedded in paraffin. The paraffin embedded sections were stained with Hematoxylin and eosin (H&E). IHC was performed as previously described^46^. Paraffin embedded sections were stained with Myeloperoxidase (1:1000, DAKO-A0398). Whole slides were scanned using an Aperio AT2 digital slide scanner (Leica Biosystems, Buffalo Grove, IL) to create slide image data files at 0.5 µm/pixel resolution. Images files were stored in eSlide Image Management System and viewed using Aperio ImageScope software (Leica Biosystems, Buffalo Grove, IL).

### Transplantation

Recipient C57BL/6 mice that expressed the CD45.1 allele of CD45 were lethally or sub-lethally irradiated (1000 cGy or 600 cGy respectively) and transplanted with donor leukemic cells, thymocytes, or BM that expressed the CD45.2 allele, as specified in the text. Engraftment of donor cells was determined by flow cytometric analyses on whole blood obtained from the tail vein as described previously^47^. Engraftment was assessed at fixed intervals after transplantation, most commonly 4, 6, 8, 12 and 16 weeks. PB samples were divided into different tubes for flowcytometry (FACS) and complete blood count (CBC). CBCs were determined using a HEMAVET Multispecies Hematology Analyzer (CDC Technologies). To evaluate BM engraftment, mice were euthanized when they showed disease symptoms (weight loss, lethargy, tachypnea, or hunched posture) and BMNC were harvested from femora and tibiae. Morphology of the PB and BM was evaluated after staining with May-Grunwald Giemsa (MGG) (MG1L-1L- SLBR9565V, GS500-500ML-SLBK4364V, Sigma Aldrich).

### RNASeq

RNA was extracted from BM of leukemic mice or lineage negative (Lin negative) purified cells from WT mice. RNA samples were prepared using TRIzol reagent (Invitrogen) following the manufacturer’s recommended protocol. Samples were evaluated using Fragment Analyzer and NanoDrop. Indexed cDNA sequencing libraries were prepared using the TruSeq Stranded mRNA Sample Preparation Kit, and barcoded with individual tags, following the manufactures recommendations. Library preparation was performed using a 96-well plate method. The libraries were quantified using Qubit 3 fluoroscopy and the NGS kit for Fragment Analyzer. Any failed results from the fragment analyzer were repeated using 2100 Bioanalyzer. Indexed libraries were prepared as equimolar pools and run on HiSeq. 4000 (2 × 150 paired end runs) to generate 40 million paired-end reads per sample library. Sequences were aligned to mm10 using STAR-2.5.3a aligner. Reads were quantified to RefSeq release 2/8/17 using Partek Flow E/M algorithm, and then normalized to fragments per kilobase of transcript per Million mapped reads (FPKM). To remove genes that had low expression across the samples, we used noise reduction filter (geometric mean = 1.0) to obtain filtered read counts. Differentially expressed genes were quantified by GSA statistical analysis method and filtered to exclude genes when less than 5% of the data values have at least a 2-fold change in either direction from the gene’s median value. Filtered genes were then used to perform hierarchical clustering and to determine differential expression by comparing BM of NP23 and NHD13 single transgenic AML samples to the WT Lin^−^BM control. Primary data available at GEO accession.

### Gene set enrichment analysis

Gene set enrichment analysis was performed using GSEA software (v 3.0) (GSEA, broadinstitute.org, MSigDB database v6.1, updated October 2017 and GSEA/MSigDB web site v6.3 released January 2018). The Hallmark and Human AML and Normal Human HSPC gene sets were obtained from MSigDB. Custom gene expression signature was generated from RNASeq data as a ranked file format and uploaded to GSEA. The ranked file of the RNASeq data were then compared to respective gene set using the GSEA preranked analysis method. Further biological relevance at the gene set level were performed with leading edge analysis, and significance of the enriched gene sets was determined by false discovery rate (FDR) values.

### Gene expression analysis using NanoString nCounter™ system

A custom designed nanostring CodeSet (PDar1), consisting of nanostring probes targeting 109 genes of interest including 6 housekeeping genes were purchased from nanostring (NanoString Technologies, Seattle, WA, USA). Probes were designed to target as many isoforms as possible for each target gene (Supplementary Table S4). 100 ng of RNA was used from each leukemic or control sample for sample preparation. Sample preparation was performed according to the manufacturer’s protocol (NanoString Technologies, Seattle, WA, USA). The nCounter XT CodeSet gene expression assay was run on a nCounter® MAX Analysis System at the NCI DNACore facility. Data generated was analyzed using nSolver 4.0 software.

### Whole exome sequencing

Libraries for whole exome capture were prepared from 50 ng genomic DNA using the Nextera DNA Library Preparation Kit (Illumina, San Diego, CA) with modifications. The mean tagmentation product size was enhanced by incubating at 58C for 10 min, the reaction terminated with the addition of 0.4% SDS, and cleaned-up using 1X SPRIselect paramagnetic beads (Thermo Fisher, Massachusetts, USA). Custom PCR primers N70x (5′-AATGATACGGCGACCACCGAGATCTACACNNNNNNNNTCGTCGGCAGCGTC-3′) and N50x (5′-CAAGCAGAAGACGGCATACGAGATNNNNNNNNGTCTCGTGGGCTCGG-3′) were substituted for the kit Index1 (i7) and Index2 (i5) primers respectively at a final concentration of 50 nM each (NNNNNNNN = Nexera index sequences). PCR products were purified using 1X SPRIselect beads (Thermo Fisher). Pooled, indexed libraries were captured using the Agilent SureSelect Mouse All Exon, 50 Mb Kit (Agilent, Santa Clara, CA) according to the manufacturer’s protocol, with the addition of xGen Custom Nextera Blocking Oligos (IDT, Illinois, USA) to the manufacturer’s specification. Libraries were sequenced on an Illumina HiSeq. 2000.Data processing and analysis was done with the NCI Genetics Branch pipeline which followed the Best Practice Variant Detection procedure recommended by the Broad Institute. Briefly, the generated raw data were mapped to mouse genome mm10 with Burrows-Wheeler Aligner (BWA) followed by local realignment using the GATK suite from the Broad Institute. Somatic variant calling was performed using the Mutect2 somatic variant caller for tumor vs. wild-type reference samples and germline variant calling was performed using UnifiedGenotyper (Broad Institute). Variants were annotated from publicly available sources. Mutations were verified by PCR amplification and Sanger sequencing of leukemic and matching non-leukemic tissue from mice with AML. Primary data available at SRA accession.

### Tcrb VDJ/DJ PCR assay and sequencing

Genomic DNA was isolated from BM, spleen and thymus using DNeasy® Blood & Tissue kit (Qiagen), following the manufacture’s recommended protocol. PCR reactions were designed to determine respective DJ rearrangements as depicted in Supplementary Fig. S2b. We designed two D primers (D1.1 and D2.2) that anneal 5′ of the D1 or D2 segment, respectively, region and two J1 primers (J1S4.2, J1S6.1) that anneal 3′ of J1S4 and J1S6 respectively, and two J2 primers (J2S4.2, J2S7.2) that anneal 3′ of J2S4 and J2S7 respectively. Primer combinations employed for PCR are indicated in Supplementary Fig. S2. Clonal bands were excised and sequenced at the NCI-NIH core facility by the Sanger method. Clonal sequences were mapped back to the mouse genome by BLAST comparison with the annotated reference sequence (Accession number MMAE000665).

DNA quality control was assessed by amplification of the single copy Scid locus (Supplementary Table S12). To detect clonal Tcrb VDJ rearrangements, RNA was isolated from leukemic BM, spleen and thymus as previously described, reverse transcribed, and amplified by PCR using a degenerative Tcrb V primer and a Tcrb C region primer, as previously described^47,48^.

### Thymocyte co-culture on OP9 and OP9-DL1 cell

OP9 or OP9-DL1 cells were maintained in culture with MEM-alpha(1X) medium (Gibco) containing 20% FBS, L-Glutamine and penicillin/streptomycin (1X) (Gibco) in a 10 cm tissue culture dish. Cells were passaged at two-day intervals (1:4). Before the day of co-culture 10^5^ cells were plated in a 12 well plate to obtain 70–80% confluency on the next day. 2–5 × 10^5^/ml DN thymocytes were then plated on OP9 or OP9-DL1 cells for *in vitro* differentiation assays in presence of myeloid (IL-3 10 ng/ml, GMCSF 10 ng/ml, SCF 10 ng/ml) and T-cell specific cytokines (IL7 1 ng/ml and Flt3 ligand 5 ng/ml)^25^.

### Statistical analysis

Data are presented as average values ± SD from individual experiments/animals as stated in figure legends. Statistical analysis was carried out using a one-way ANOVA with a correction provided by the Tukey’s multiple comparisons test, or one-tailed unpaired t test for three or fewer samples and a Mann-Whitney test for four and greater than four samples, using GraphPad Prism software-7. *P* values < 0.05 were considered as statistical significance.

## Supplementary information

Supplementary information

Supplementary tables 3,4,5

## References

[1] Orkin SH, Zon LI (2008). Hematopoiesis: an evolving paradigm for stem cell biology. Cell.

[2] Seita J, Weissman IL (2010). Hematopoietic stem cell: self-renewal versus differentiation. Wiley Interdiscip Rev Syst Biol Med.

[3] Bryder D, Rossi DJ, Weissman IL (2006). Hematopoietic stem cells: the paradigmatic tissue-specific stem cell. Am J Pathol.

[4] Mikkola HK, Orkin SH (2006). The journey of developing hematopoietic stem cells. Development.

[5] Bhandoola A, von Boehmer H, Petrie HT, Zuniga-Pflucker JC (2007). Commitment and developmental potential of extrathymic and intrathymic T cell precursors: plenty to choose from. Immunity.

[6] Ceredig R, Bosco N, Rolink AG (2007). The B lineage potential of thymus settling progenitors is critically dependent on mouse age. Eur J Immunol.

[7] Balciunaite G, Ceredig R, Rolink AG (2005). The earliest subpopulation of mouse thymocytes contains potent T, significant macrophage, and natural killer cell but no B-lymphocyte potential. Blood.

[8] Plaks V, Kong N, Werb Z (2015). The cancer stem cell niche: how essential is the niche in regulating stemness of tumor cells?. Cell Stem Cell.

[9] Kreso A, Dick JE (2014). Evolution of the cancer stem cell model. Cell Stem Cell.

[10] Armstrong SA (2002). MLL translocations specify a distinct gene expression profile that distinguishes a unique leukemia. Nat Genet.

[11] Zhou HS, Carter BZ, Andreeff M (2016). Bone marrow niche-mediated survival of leukemia stem cells in acute myeloid leukemia: Yin and Yang. Cancer Biol Med.

[12] Pineault N (2003). Induction of acute myeloid leukemia in mice by the human leukemia-specific fusion gene NUP98-HOXD13 in concert with Meis1. Blood.

[13] Gough SM (2014). NUP98-PHF23 is a chromatin-modifying oncoprotein that causes a wide array of leukemias sensitive to inhibition of PHD histone reader function. Cancer Discov.

[14] Arai Y (2000). Heterogenous fusion transcripts involving the NUP98 gene and HOXD13 gene activation in a case of acute myeloid leukemia with the t(2;11)(q31;p15) translocation. Leukemia.

[15] Heinrichs S (2005). CD34+ cell selection is required to assess HOXA9 expression levels in patients with myelodysplastic syndrome. Br J Haematol.

[16] Lin YW, Slape C, Zhang Z, Aplan PD (2005). NUP98-HOXD13 transgenic mice develop a highly penetrant, severe myelodysplastic syndrome that progresses to acute leukemia. Blood.

[17] Slape C., Lin Y. W., Hartung H., Zhang Z., Wolff L., Aplan P. D. (2008). NUP98-HOX Translocations Lead to Myelodysplastic Syndrome in Mice and Men. JNCI Monographs.

[18] Reader JC, Meekins JS, Gojo I, Ning Y (2007). A novel NUP98-PHF23 fusion resulting from a cryptic translocation t(11;17)(p15;p13) in acute myeloid leukemia. Leukemia.

[19] Gough SM (2017). Progenitor B-1 B-cell acute lymphoblastic leukemia is associated with collaborative mutations in 3 critical pathways. Blood Adv.

[20] Kogan SC (2002). Bethesda proposals for classification of nonlymphoid hematopoietic neoplasms in mice. Blood.

[21] Choi CW, Chung YJ, Slape C, Aplan PD (2009). A NUP98-HOXD13 fusion gene impairs differentiation of B and T lymphocytes and leads to expansion of thymocytes with partial TCRB gene rearrangement. J Immunol.

[22] Rothenberg EV, Taghon T (2005). Molecular genetics of T cell development. Annu Rev Immunol.

[23] Rothenberg EV, Moore JE, Yui MA (2008). Launching the T-cell-lineage developmental programme. Nat Rev Immunol.

[24] Rothenberg EV (2014). Transcriptional control of early T and B cell developmental choices. Annu Rev Immunol.

[25] Bell JJ, Bhandoola A (2008). The earliest thymic progenitors for T cells possess myeloid lineage potential. Nature.

[26] Holmes R, Zuniga-Pflucker JC (2009). The OP9-DL1 system: generation of T-lymphocytes from embryonic or hematopoietic stem cells *in vitro*. Cold Spring Harb Protoc.

[27] Porritt HE (2004). Heterogeneity among DN1 prothymocytes reveals multiple progenitors with different capacities to generate T cell and non-T cell lineages. Immunity.

[28] Pietras EM (2015). Functionally Distinct Subsets of Lineage-Biased Multipotent Progenitors Control Blood Production in Normal and Regenerative Conditions. Cell Stem Cell.

[29] Tan C (2011). Ten-color flow cytometry reveals distinct patterns of expression of CD124 and CD126 by developing thymocytes. BMC Immunol.

[30] Maynadie M (2011). Twenty-five years of epidemiological recording on myeloid malignancies: data from the specialized registry of hematologic malignancies of Cote d’Or (Burgundy, France). Haematologica.

[31] Hirouchi T (2008). Upregulation of c-myc gene accompanied by PU.1 deficiency in radiation-induced acute myeloid leukemia in mice. Exp Hematol.

[32] Turk V, Bode W (1991). The cystatins: protein inhibitors of cysteine proteinases. FEBS Lett.

[33] Ericson JA (2014). Gene expression during the generation and activation of mouse neutrophils: implication of novel functional and regulatory pathways. PLoS One.

[34] Del Real MM, Rothenberg EV (2013). Architecture of a lymphomyeloid developmental switch controlled by PU.1, Notch and Gata3. Development.

[35] Yui MA, Rothenberg EV (2014). Developmental gene networks: a triathlon on the course to T cell identity. Nat Rev Immunol.

[36] Riemke P (2016). Myeloid leukemia with transdifferentiation plasticity developing from T-cell progenitors. EMBO J.

[37] Lee JY (2017). A case of synchronous multiple myeloma and chronic myeloid leukemia. Blood Res.

[38] Maschmeyer G, Brink I, Jahne D, Arnold R, Schega O (2017). Residual thymic tissue and lymph node involvement by acute myeloid leukaemia presenting as mediastinal, strongly (18) FDG-PET-positive masses. Eur J Haematol.

[39] Astall E, Yarranton H, Arno J, Marcus R (1999). Granulocytic sarcoma preceding AML M0 and the diagnostic value of CD34. J Clin Pathol.

[40] Chubachi A (1993). Acute myelogenous leukemia associated with a mediastinal tumor. Leuk Lymphoma.

[41] Ramasamy K (2007). Acute myeloid leukaemia presenting with mediastinal myeloid sarcoma: report of three cases and review of literature. Leuk Lymphoma.

[42] Savage NM (2010). Acute leukemia with PICALM-MLLT10 fusion gene: diagnostic and treatment struggle. Cancer Genet Cytogenet.

[43] Asnafi V (2003). CALM-AF10 is a common fusion transcript in T-ALL and is specific to the TCRgammadelta lineage. Blood.

[44] Dik WA (2005). CALM-AF10+ T-ALL expression profiles are characterized by overexpression of HOXA and BMI1 oncogenes. Leukemia.

[45] Caudell D, Aplan PD (2008). The role of CALM-AF10 gene fusion in acute leukemia. Leukemia.

[46] Beachy SH (2012). Enforced expression of Lin28b leads to impaired T-cell development, release of inflammatory cytokines, and peripheral T-cell lymphoma. Blood.

[47] Chung YJ, Choi CW, Slape C, Fry T, Aplan PD (2008). Transplantation of a myelodysplastic syndrome by a long-term repopulating hematopoietic cell. Proc Natl Acad Sci USA.

[48] Danska JS (1994). Rescue of T cell-specific V(D)J recombination in SCID mice by DNA-damaging agents. Science.

