# Supplementary material for: Thymic precursor cells generate acute myeloid leukemia in NUP98-PHF23/NUP98-HOXD13 double transgenic mice
Source: Sci Rep. 2019 Nov 20;9:17213. doi: 10.1038/s41598-019-53610-7 (PMC6868234; doi:10.1038/s41598-019-53610-7)
Supplement: Supplementary file 1 — Supplementary information [file 41598_2019_53610_MOESM1_ESM.pdf]

Thymic precursor cells generate acute myeloid leukemia in NUP98-PHF23/NUP98-HOXD13 double transgenic mice

Subhadip Kundu, Eun Sil Park, Yang Jo Chung, Robert L. Walker, Yuelin J. Zhu, Vijay Negi, Paul S. Meltzer, Peter D. Aplan

Supplementary information included in pdf.

Supplementary Figures 1-16

Supplementary Tables 1-2, 6-12.

Supplementary Tables 3-5 are attached separately as sortable Excel files.

Figure S1

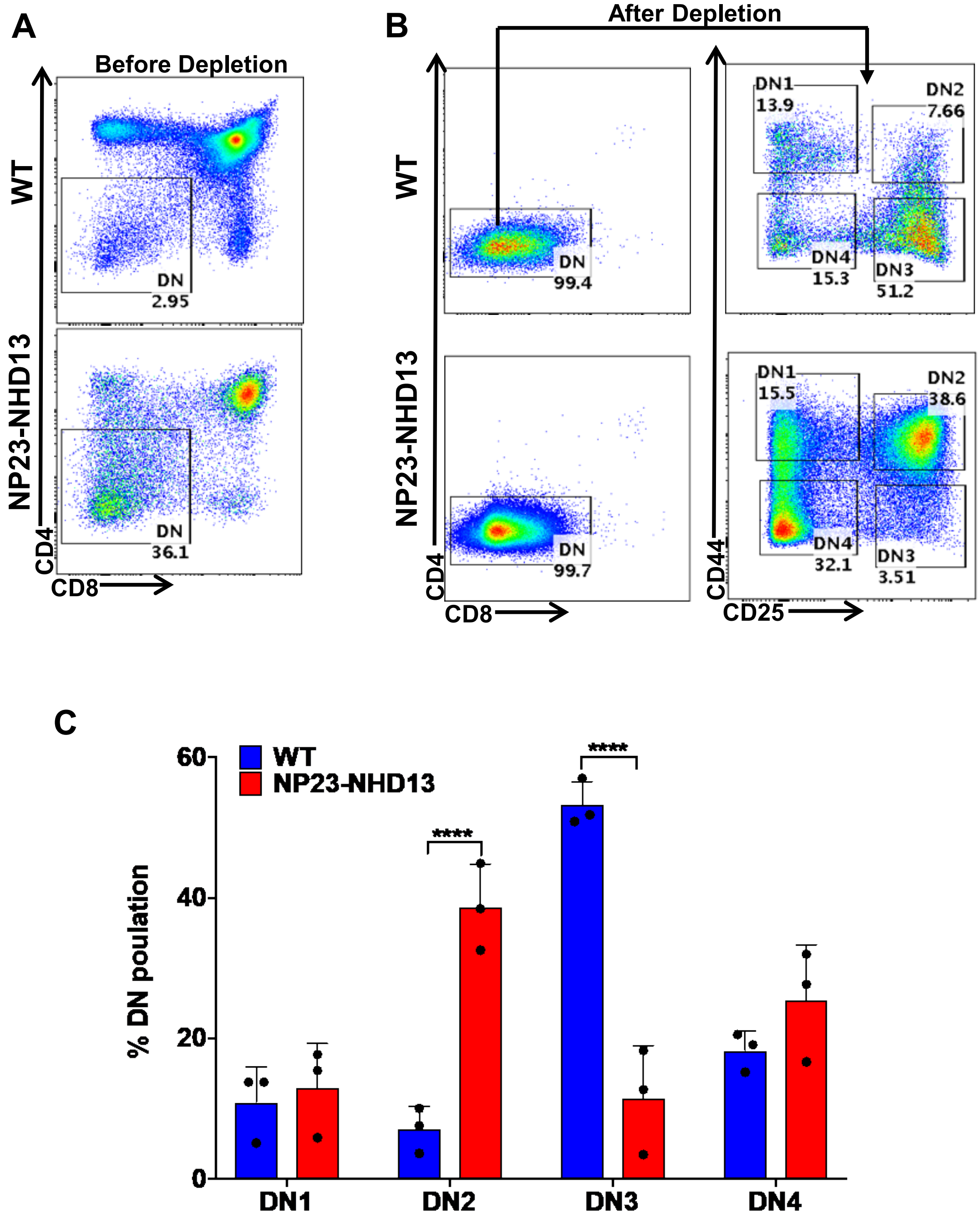

Figure S1. Thymic differentiation is impaired in young, non-leukemic NP23-NHD13 mice. (A) Flow cytometry of young (22-30 days) WT and NP23-NHD13 thymus before depletion of DP and SP CD4 and CD8 cells. **(B)** Flow cytometry of WT and NP23-NHD13 thymus after depletion of DP and SP CD4 and CD8 cells. **(C)** Fractionated DN sub-populations (DN1, DN2, DN3 and DN4). Data are expressed as means ± SD, *n*=3 mice. *****P*<0.001, by one tailed student t-test.

Figure S2.

A

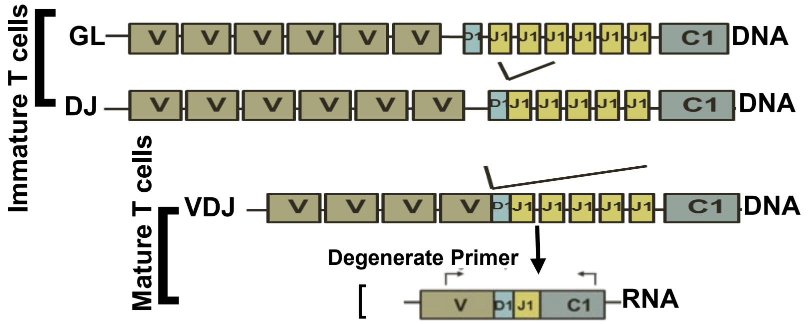

B

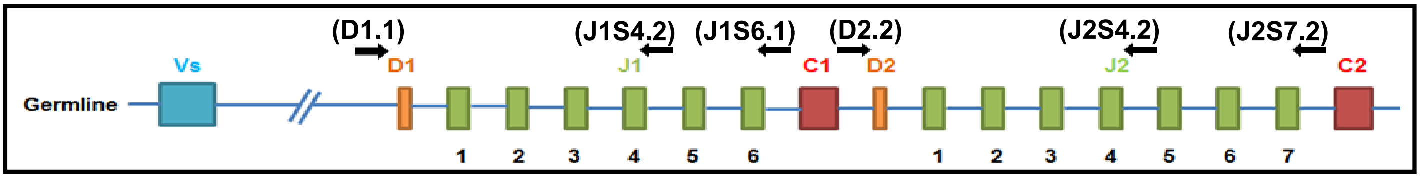

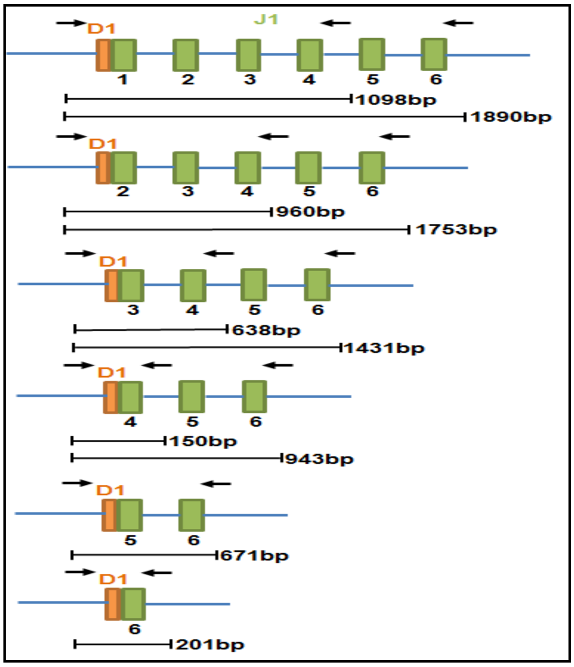

Rearrangement	D1.1/JIS4.2 Primers (PCR Product Size)	D1.1/JIS6.1 Primers (PCR Product Size)
No Rearrangement (Germline)	1750bp	2524bp
D1-J1S1	1098bp	1890bp
D1-J1S2	960bp	1753bp
D1-J1S3	638bp	1431bp
D1-J1S4	150bp	943bp
D1-J1S5	No PCR Product	671bp
D1-J1S6	No PCR Product	201bp

Rearrangement	D2.2/J2S4.2 Primers (PCR Product Size)	D2.2/J2S7.2 Primers (PCR Product Size)
No Rearrangement (Germline)	1329bp	1830bp
D2-J2S1	751bp	1253bp
D2-J2S2	548bp	1050bp
D2-J2S3	281bp	783bp
D2-J2S4	142bp	644bp
D2-J2S5	No PCR Product	553bp
D2-J2S6	No PCR Product	408bp
D2-J2S7	No PCR Product	196bp

C

Mouse ID	Tcrb-DJ			Tcrb-VDJ		
	BM	Spleen	Thymus	BM	Spleen	Thymus
M4318	GL	GL	GL	NO	NO	NO
M4324	GL	GL	GL	NO	NO	NO
M4351	GL	GL	GL	NO	NO	NO
M4352	GL	GL	Clonal(D1J1S3)	NO	NO	NO
M4361	GL	GL	GL	NO	NO	NO
M4360	GL	GL	Clonal(D1J1S4)	NO	NO	NO
M4369	GL	GL	GL	NO	NO	NO

Figure S2. Schematic representation of Tcrb gene rearrangements and PCR strategies to determine clonal D-J rearrangement. (A) Schematic representation of Tcrb-DJ and VDJ rearrangements during thymocyte development. **(B)** Figure indicates position of the primers (D1.1, J1S4.2, J1S6.1; D2.2, J2S4.2, J2S7.2) used to amplify regions of the Tcrb locus and potential PCR product sizes. **(C)** Table indicates specific Tcrb-DJ rearrangements found in different leukemic mice, none of the primary leukemic samples showed Tcrb-VDJ rearrangements (NO=no Tcrb VDJ rearrangements detected).

Figure S3

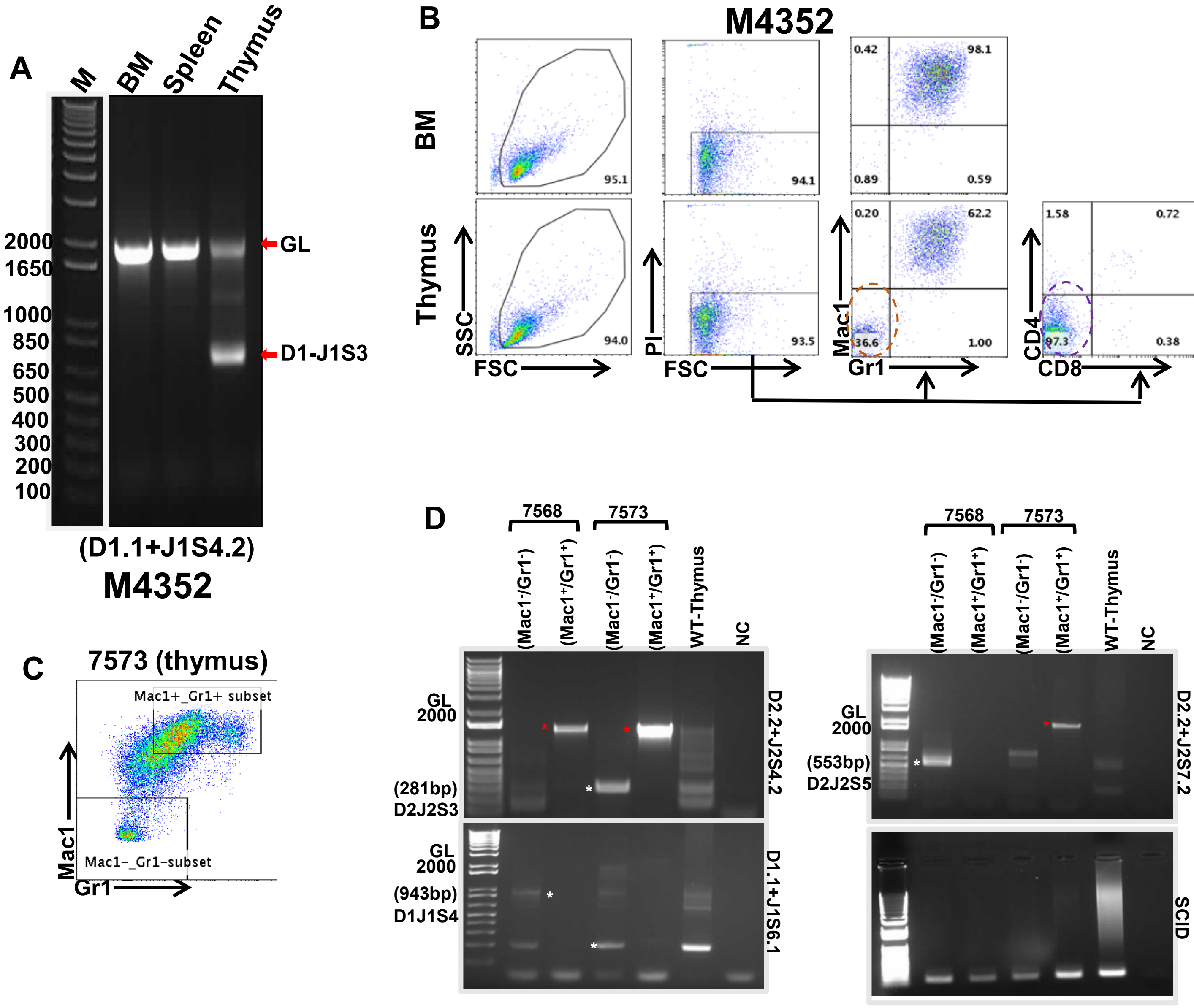

E

Mice ID/cell population	D	N	J
7568 (Mac1$^-$/Gr1$^-$)	TCRB-D1-GGGACAGGGggc	AC	ttTCCAACGAAAGATTATTTCG-J1S4
7573 (Mac1$^-$/Gr1$^-$)<br>7573 (Mac1$^-$/Gr1$^-$)	TCRB-D1-GGGACAGGGGGc<br>TCRB-D2-GGGACTGGGGGGGC	GAGGGATA<br>AGCG	ttccTATAATTCGCCCCTCTACTT-J1S6<br>agtGCAGAAACGCTGTATTTTGG-J2S3

Figure S3. Clonal Tcrb-DJ rearrangements in the Mac1$^-$/Gr1$^-$ fraction of leukemic NP23-NHD13 thymus. **(A)** A clonal Tcrb-DJ rearrangement (D1J1S3) is shown in NP23-NHD13 (#4352) AML; note that BM shows only the germline DJ fragment, not the clonal D1-J1S3 rearrangement, whereas the thymus shows a clonal D1-J1S3 rearrangement. Unrelated lanes between the size standard marker (M) and samples have been removed. **(B)** Gating strategy for flow cytometry of primary leukemic mouse demonstrating myeloid (BM and thymus) and non-myeloid (thymus) populations in mouse 4352, propidium iodide (PI) is used to discriminate live and dead cells. **(C)** Mac1$^+$/Gr1$^+$ and Mac1$^-$/Gr1$^-$ populations, sorted from NP23-NHD13 #7573 for Tcrb-DJ clonality assay. **(D)** PCR amplification demonstrates specific DJ rearrangements (indicated by white asterisk) in Mac1$^-$/Gr1$^-$ fraction, whereas no rearrangements are found in Mac1$^+$/Gr1$^+$ fraction. Germline (GL) PCR products are indicated with a red asterisk. Amplification of the *scid* locus is used as a DNA quality control indicator. **(E)** Table of clonal rearrangements in the Mac1$^-$/Gr1$^-$ fraction from two different NP23-NHD13 thymi.

Figure S4

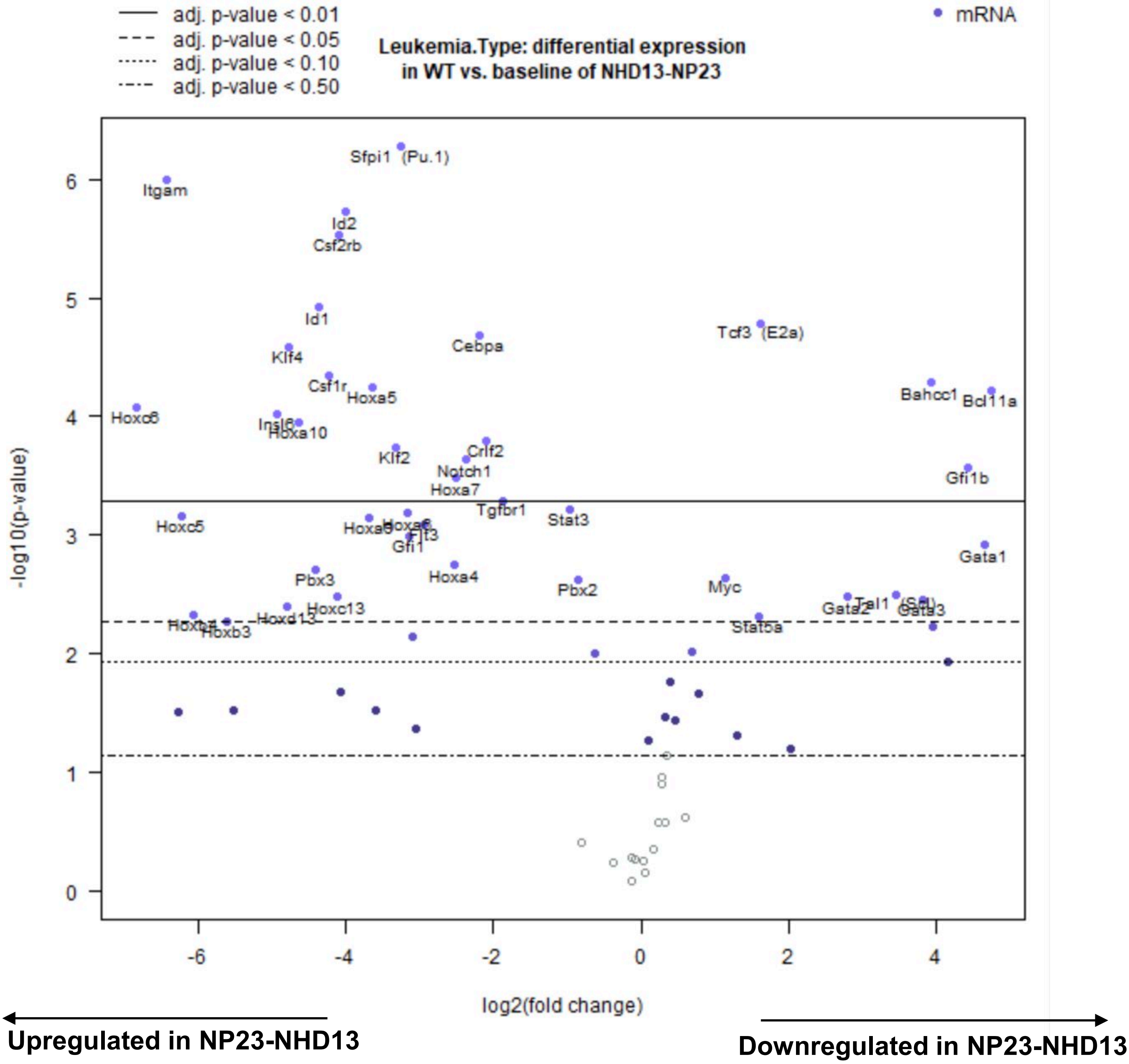

Figure S4. Gene expression assessed by nanostring technology. "Volcano" plot from nanostring assay identifies genes significantly up or down-regulated in NP23-NHD13 AML samples compared with WT Lin- BM. Log 2 (fold change) is on x axis, and -log10 (p value) is on y axis.

Figure S5

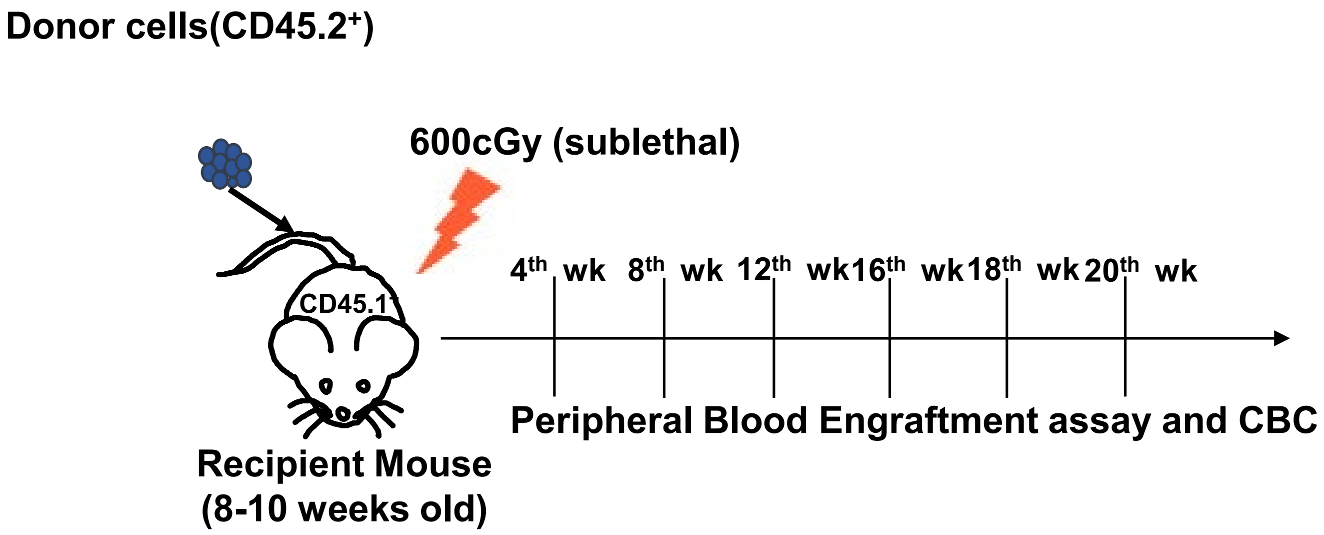

Figure S5. Strategy employed for transplantation experiments.

Figure S6

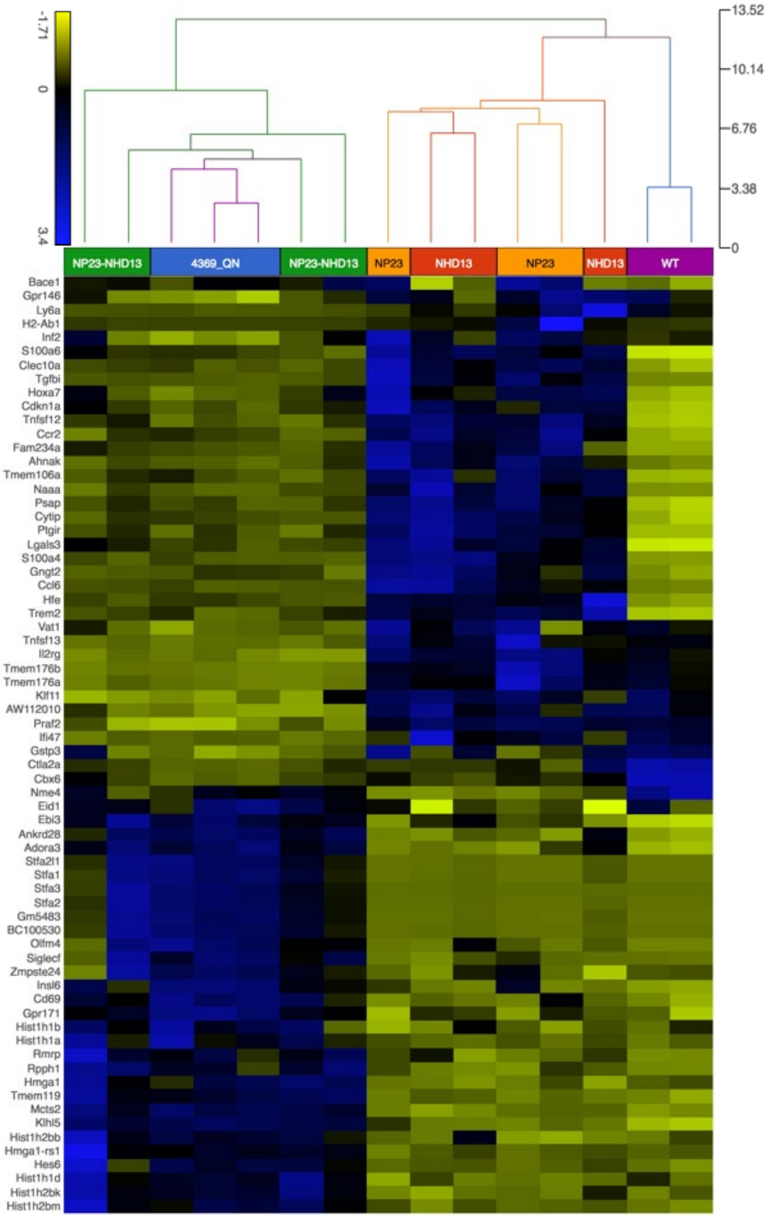

Figure S6. AML samples from QN transplanted recipients cluster with AML samples from primary NP23-NHD13 leukemic mice. Hierarchical clustering of #4369 QN transplant recipients (blue) cluster with primary NP23-NHD13 leukemia samples (green).

Figure S7

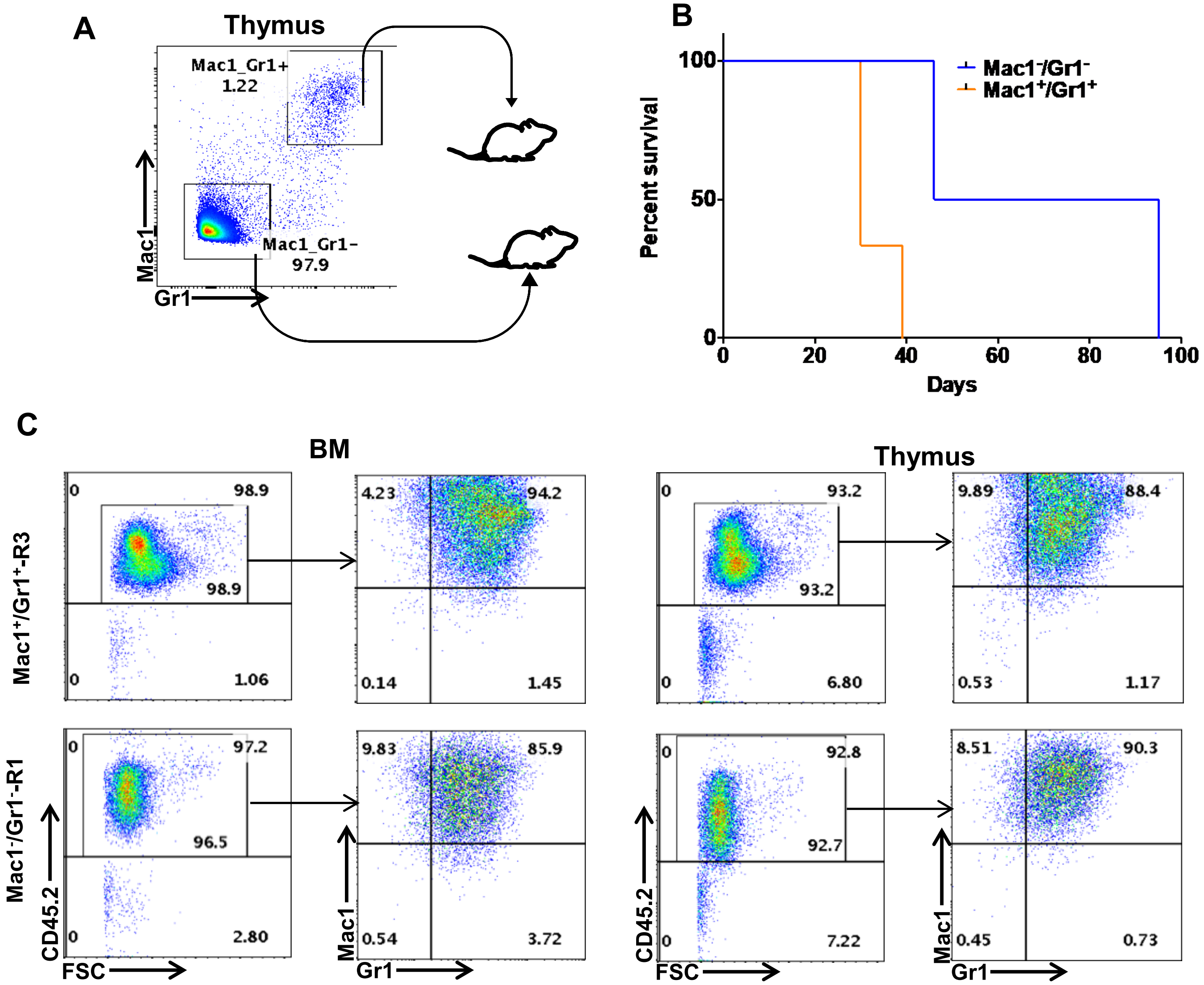

Figure S7. The Mac1$^+$/Gr1$^+$ population can also transmit AML. (A) FACS sorting of Mac1$^+$/Gr1$^+$ and Mac1$^-$/Gr1$^-$ populations from NP23-NHD13 thymus, 1x10^4 cells were transplanted to each recipient, *n*=2-3 mice per group (one Mac1-Gr1- recipient died within 2 weeks of transplant and was excluded from analysis). **(B)** Survival curve; data analyzed by Log-rank (Mantel-Cox) test; *P*=0.054. **(C)** Flow cytometry of recipient indicates that both Mac1$^+$/Gr1$^+$ and Mac1$^-$/Gr1$^-$ cells engraft and generate AML(CD45.2$^+$/Mac1+Gr1+).

Figure S8.

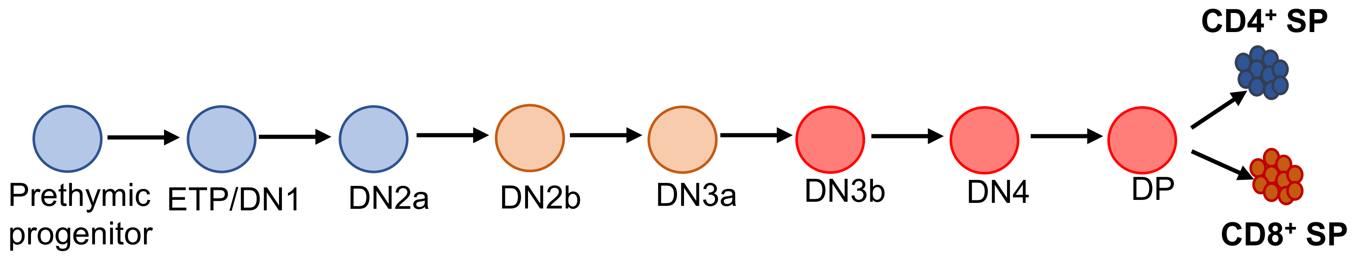

Figure S8. Schematic representation of thymocyte differentiation. Thymocyte differentiation from early T-cell precursors (ETP) to mature $CD4^{+}$ and $CD8^{+}$ thymocytes, adapted from Yui et al., 2014.

Figure S9

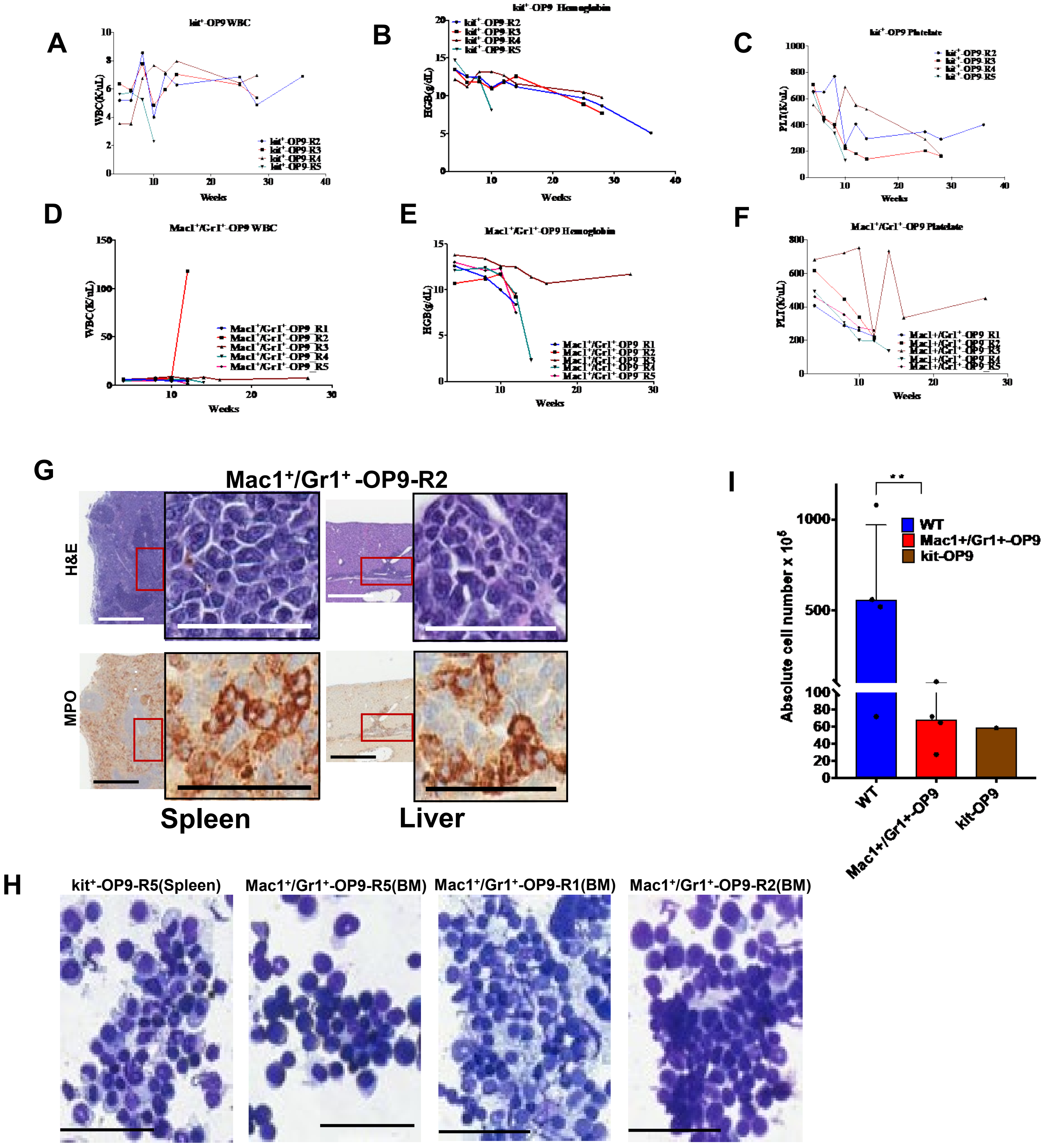

Figure S9. Kit+-OP9 and Mac1+/Gr1+ cells from OP9 co-culture develop progressive pancytopenia. (A-F) Serial blood analysis of the Kit+-OP9 and Mac1+/Gr1+ cells transplant recipients. **(G)** H & E staining depicts leukemic cell invasion in parenchymal organ (Spleen and liver) of representative Mac1+/Gr1+ recipient mouse. Myeloperoxidase (MPO)staining confirms the presence of myeloid cell population in the invaded tissues. **(H)** Blast count from (Mac1+/Gr1+ (BM) and Kit+-OP9 (Spleen) cells transplanted recipients. (**I**) Total number of BMNC harvested from 2 femora and 2 tibiae. Spleen was used for blast count in Kit+-OP9 transplant recipient. Scale bars=800μm (low power view), 50μm (high power view).

Figure S10

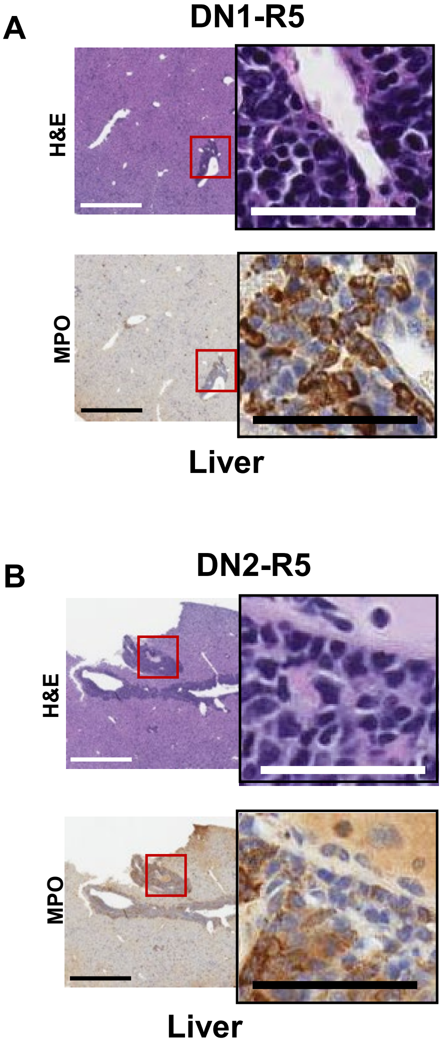

Figure S10. Histology and Immunohistochemistry confirms tissue invasion of leukemic cells. (A and B) H & E staining depicts characteristic perivascular/periportal leukemic cell invasion in liver of representative DN1 and DN2 recipient with AML. Myeloperoxidase staining confirms the presence of myeloid cell population in the invaded tissues. Scale bars=800μm (low power view), 50μm (high power view).

Figure S11

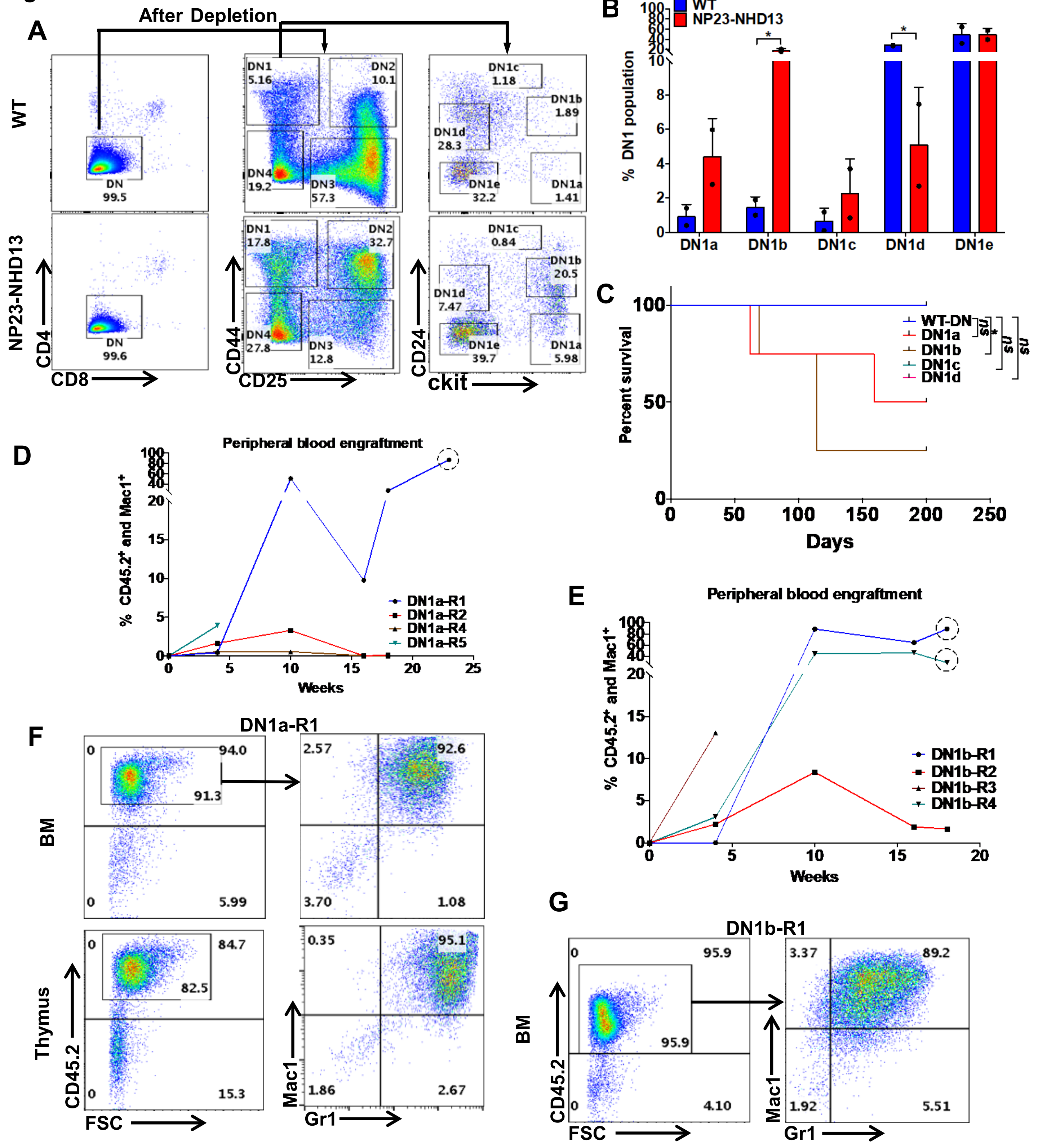

Figure S11. DN1a and DN1b populations are increased in NP23-NHD13 thymocytes and transmit AML. (A) FACS sorting and transplantation of DN1 subpopulation from NP23-NHD13 mice (DN1a=120 cells/mouse, DN1b=400 cells/mouse, DN1c=11cells/mouse, DN1d=114 cells/mouse, (DN1e were also transplanted and did not show engraftment at week 10, (however most of the mice in this group were euthanized because of wounds from penis bites and, therefore, excluded from the study). **(B)** Bar graph indicates increased DN1b and decreased DN1d sub-population, Data are expressed as means ± SD, *n*=2 mice per group. **(C)** Survival curve indicates DN1a and DN1b recipients develop disease, *n*=4-5 mice per group. Data analyzed by Log-rank (Mantel-Cox) test. Serial engraftment of donor cells ($CD45.2^+$) in DN1a **(D)** and DN1b **(E)** recipients (dotted circle indicates engraftment data obtained from BM). **(F and G)** Flow cytometry indicates presence of donor-derived ($CD45.2^+$) AML cells in DN1a and DN1b recipients. $^*P<0.05$.

Figure S12

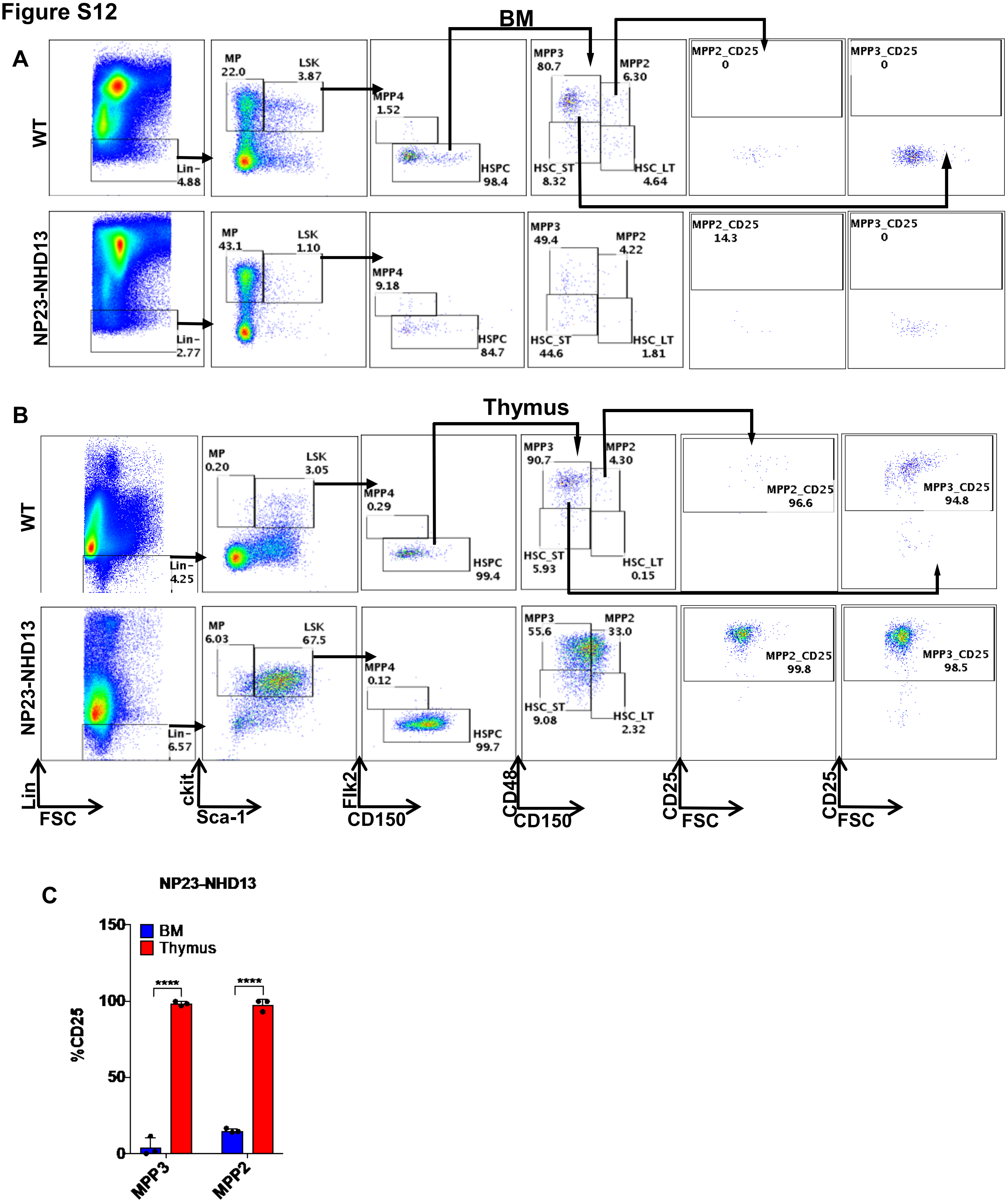

Figure S12. Identification of CD25 positive MPP3 and MPP2 populations in NP23-NHD13 thymus. (A) Analysis of CD25 expression in LSK fractions from NP23-NHD13 and WT BM. **(B)** Analysis of CD25 expression in LSK fraction from NP23-NHD13 and WT thymus. **(C)** Quantification of MPP3 and MPP2 CD25 populations in BM and thymus. Data are expressed as means ± SD, *n*=3 mice per group, ****P<0.001 by one tailed student t-test.

Figure S13

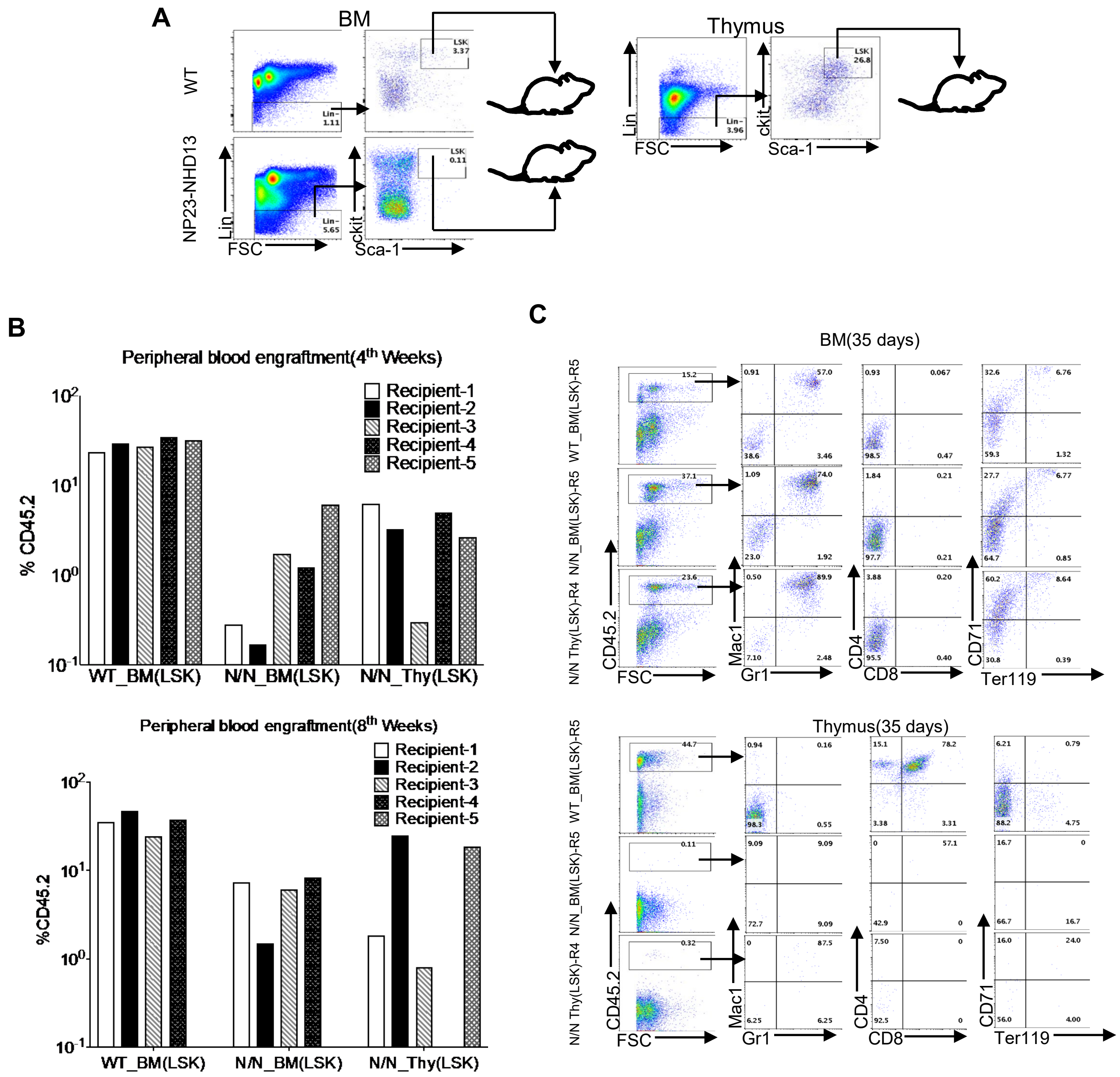

Figure S13. Thymic resident LSK cells engraft WT recipient mice. (A) Gating strategy for cell purification. WT_BM(LSK)(1420 cells/recipient), N/N_BM(LSK)(158 cells/recipient), N/N_Thy(LSK)(5136 cells/recipient). **(B)** Initial engraftment at 4th and 8th week. **(C)** Flow cytometry of BM and thymus from a representative mouse in each group at 5th week.

Figure S14

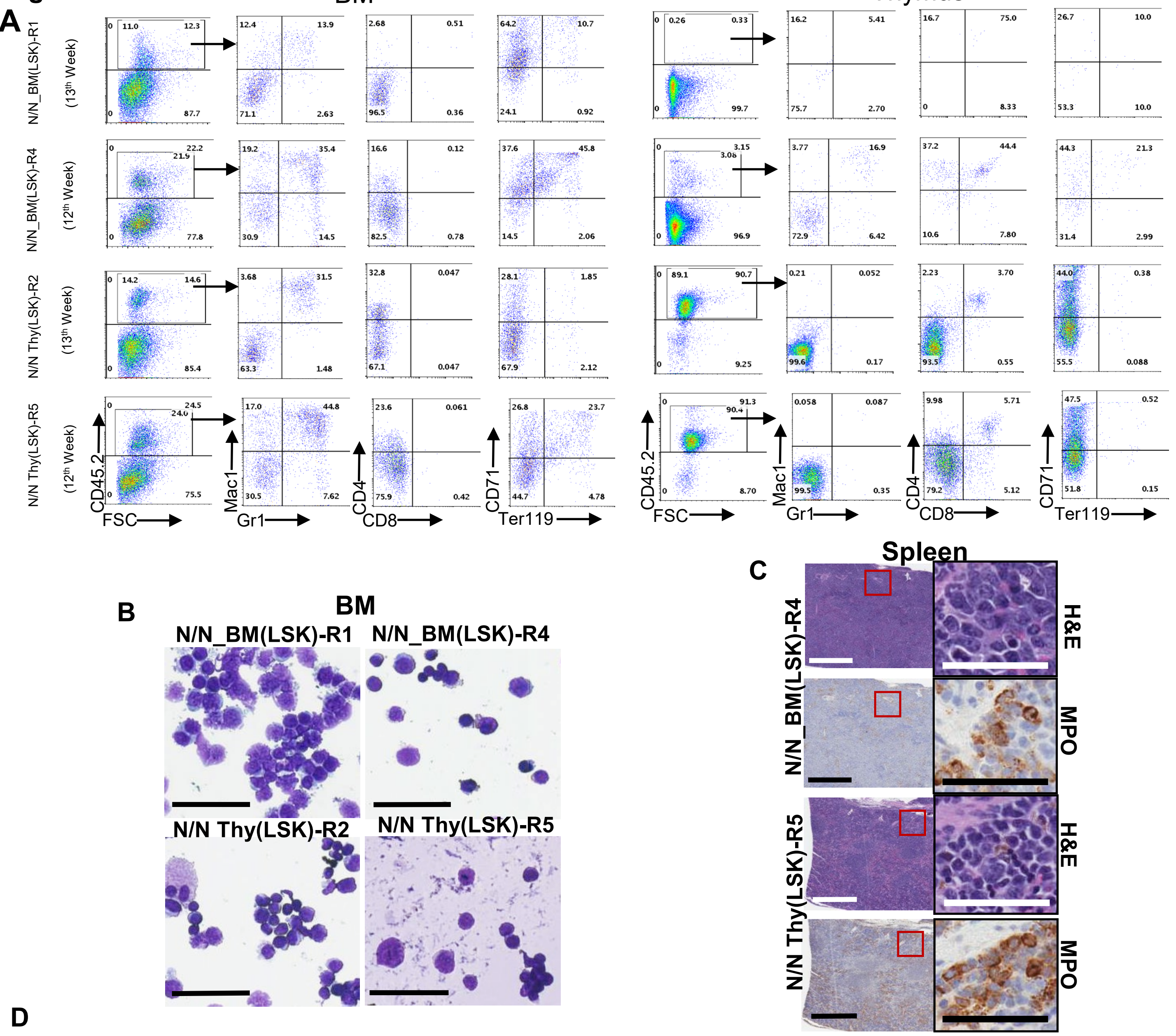

D

NP23-NHD13 Donor	Weeks (Post Transplantation)	CBC(at time of death)				
		WBC (K/uL)	PLT (K/uL)	Hb (g/dL)	BM Cellularity (2F2T)*	Blast%
NP23-NHD13-BM (LSK) Recipient-1	13th	16.58†	126	4.10	$5.4x10^7$	98%
NP23-NHD13-BM (LSK) Recipient-4	12th	ND	ND	ND	$1.55x10^7$	98%
NP23-NHD13-Thy (LSK) Recipient-2	13th	108.48†	301	5.90	$5.1x10^7$	98%
NP23-NHD13-Thy (LSK) Recipient-5	12th	6.16	248	8.90	$1.43x10^7$	99%

Figure S14. Thymic resident LSK cells can transmit AML. (A) Flow cytometry profile of leukemic recipients of LSK cells from NP23-NHD13 BM (upper two panels) or thymus (lower two panels). **(B)** Blast morphology in BM of recipients that developed AML **(C)** H &E and myeloperoxidase (MPO) staining demonstrate infiltration of MPO positive cells. **(D)** CBC and blast percentage at the time of death. N/N= NP23-NHD13 mice. Scale bars=800μm (low power view), 50μm (high power view). ND (Not Determined), † WBC for recipients indicate high cell count,* Indicates cell counts from two femur and two tibia.

Figure S15

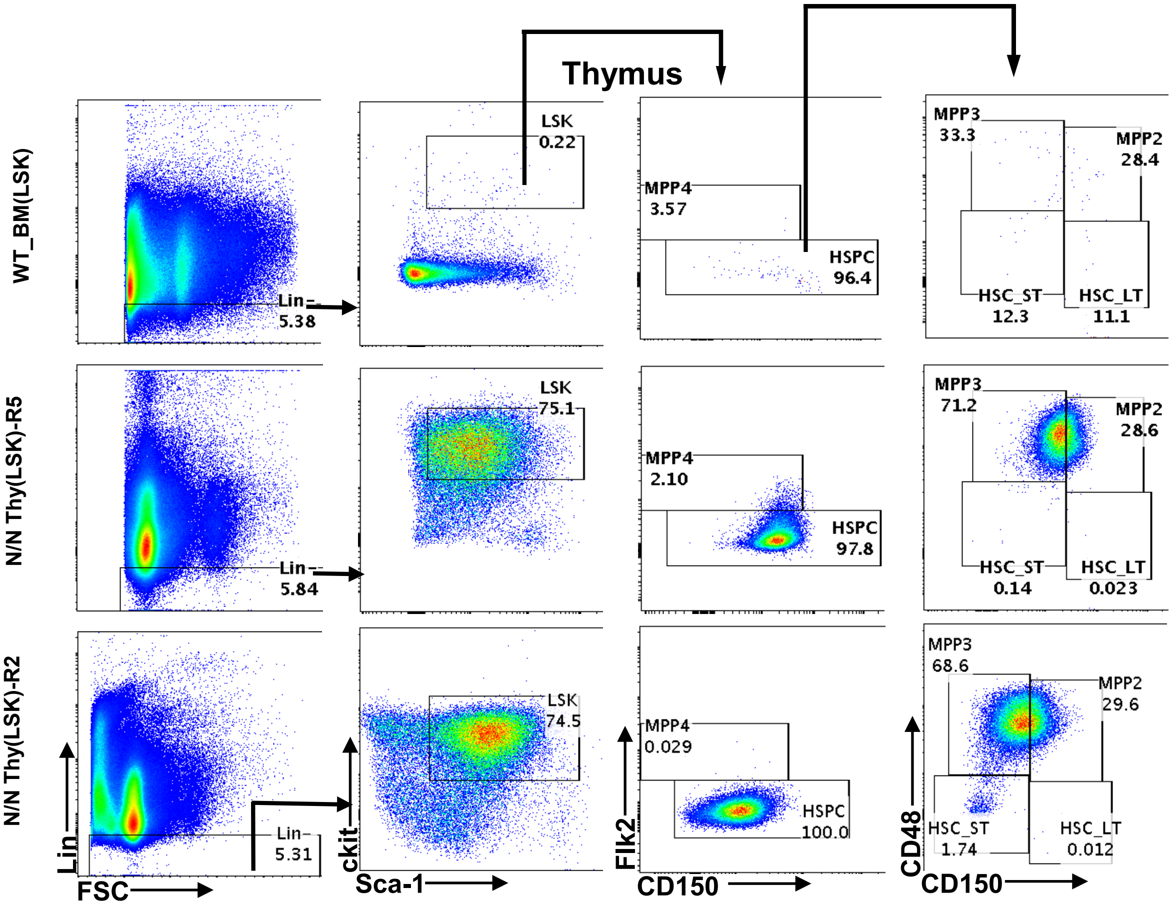

Figure S15. Thymic resident LSK cells from NP23-NHD13 mice migrate to the thymus of transplant recipients. Thymic LSK cells from N/N_Thy (LSK) donor migrates to the thymus of the transplant recipients (N/N_Thy(LSK) recipients R2 and R5) and produce a homogenous MPP2/3 population. N/N= NP23-NHD13 mice.

Figure S16

A

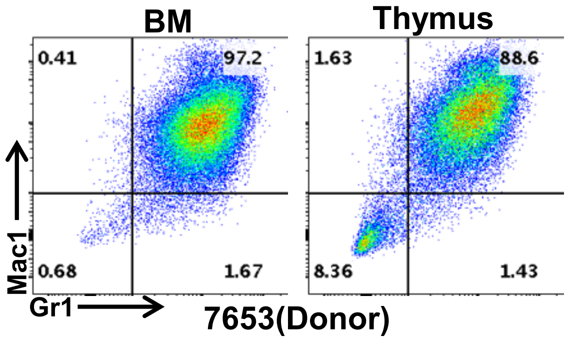

B

Cell Transplanted (NP23-NHD13 Leukemic mouse)	Dose (Cell No injected)	Tested (No of Mice)	Response (engraftment)
**Bone Marrow**	40	4	1
	100	4	2
	1000	2	1
**Thymus**	40	3	2
	100	2	2
	1000	4	4

Range of LIC from thymus and BM estimated at 1:32(range 10.2-103) and 1:441(range 123-1585) respectively using online tool available at

http://bioinf.wehi.edu.au/software/elda/.

Figure S16. LIC are more frequent in thymus than BM. (A) Flow cytometry of BM and thymus from leukemic donor mouse. (B) engraftment of 40, 100, or 1000 cells injected into 2-4 sublethally irradiated recipients.

Thymic precursor cells generate acute myeloid leukemia in NUP98-PHF23/NUP98-HOXD13 double transgenic mice

Subhadip Kundu, Eun Sil Park, Yang Jo Chung, Robert L. Walker, Yuelin J. Zhu, Vijay Negi, Paul S. Meltzer, Peter D. Aplan

Supplementary information included in pdf.

Supplementary Figures 1-16

Supplementary Tables 1-2, 6-12.

Supplementary Tables 3-5 are attached separately as sortable Excel files.

Supplementary Table 1. Detailed characterization of NP23-NHD13 leukemic mice

ID	Sex	Age, Days	CBC WBC (K/uL)	PLT (K/uL)	Hb (g/dL)	LY (K/uL)	Polys (K/uL)	Mono (K/uL)	IHC pathology	Myeloperoxidase	Flow Cytometry(%)	Mac1+/Gr1+	B220+	CD4+/CD8+	Diagnosis
									Spleen	+	BM	95.83	0	0.00	
4318									Liver	+	Spleen	46.76	0.05	0.00	AML
	F	69	High†	765	7.5	80.46	High	98.87	Thymus	++	Thymus	95.76	0.30	0.00	
									Spleen	+	BM	87.84	1.53	0	
4324*									Liver	+	Spleen	42.55	1.16	0.02	AML
	M	67	568.32	2508	10.8	256.72	204.92	78.64	Kidney	+	Thymus	98.45	1.45	0	
									Spleen	++	BM	98.36	ND	0.30	
4351									Liver	+	Spleen	42.9	ND	1.63	AML
	F	68	118.2	658	8.10	40.52	45.80	26.90	Kidney	+	Thymus	87.1	ND	0.59	
									Spleen	++++	BM	98.19	0.14	0.00	
4352									Liver	+++	Spleen	81.81	2.04	0.04	AML
	F	40	32.82	387	6.90	20.13	6.55	5.55	Thymus	++	Thymus	62.73	1.93	0.84	
									Spleen	++++	BM	56.18	1.24	0.34	
4361*									Liver	+++	Spleen	10.21	0.96	0.13	AML
	F	51	High†	1118	8.20	High	High	High	Thymus	++++	Thymus	85.83	0.7	0.14	
									Spleen	++++	BM	83.59	0.88	0	
4360									Liver	+++	Spleen	41.74	2.55	0.28	AML
	F	56	525.04	1994	10.4	138	284.32	68.88	Thymus	++++	Thymus	64.05	0.02	0.14	
									Spleen	++	BM	44.91	1.51	0.57	
4369*									Liver	+++	Spleen	6.81	1.14	0.02	AML
	F	51	171.74	643	4.2	112.82	38.09	17.48	-		Thymus	84.89	ND	0.14	
									Spleen	++	BM	85.7	0.54	0.04	
1283*									Liver	+++	Spleen	31.3	0.68	0.13	AML
	F	67	444.500	312	3.9	324.485	45.339	1.334	Kidney	+++	Thymus	87.8	0.54	0.10	
									Spleen	++++	BM	91.5	1.36	0.01	
1286*									Liver	++	Spleen	36.3	0.33	0.06	AML
	F	50	ND	ND	ND	ND	ND	ND	Kidney	+++	Thymus	95.3	1.00	0.13	

AML (acute myeloid leukemia); CBC (complete blood count).
+/++ (moderate expression); +++/++++ (high expression).
† WBC for samples 4361 and 4318 were out of range for the instrument and reported as "High".
** Indicates mice in which thymic invasion was greater than BM.*
ND (Not Determined).

Supplementary Table 2. CBC and flow cytometry of young WT and NP23-NHD13 mice

ID	Sex	Age, Days	CBC			Flow Cytometry					
			WBC (K/uL)	PLT (K/uL)	Hb (g/dL)	Mac1+ (K/uL)	Mac1+/Gr1+ (K/uL)	Gr1+ (K/uL)	CD19+/B220+ (K/uL)	CD4+ (K/uL)	CD8+ (K/uL)
7645(WT)	F	36	9.62	757.00	12.40	0.464646	0.204906	0.207792	5.5796	0.706108	0.97162
7659(WT)	M	30	12.68	979.00	12.70	0.4438	0.486912	0.656824	5.05932	1.81324	1.28068
7692(WT)	F	36	12.24	845.00	14.70	1.33416	0.604656	0.454104	5.61816	1.88496	1.7136
7653(NP23-NHD13)	M	33	4.20	571.00	11.30	0.504	1.2558	0.02016	0.0135	0.0693	0.0714
7660(NP23-NHD13)	M	30	5.46	1070.00	10.30	0.11739	2.14578	0.10374	0.020202	0.115752	0.068796
7661(NP23-NHD13)	M	30	3.08	485.00	10.90	0.08778	1.0318	0.051744	0.068068	0.06622	0.018172

Supplementary Table. 6 CBC from QN cells transplant recipients

Leukemic Donor	CBC					
	WBC (K/uL)	PLT (K/uL)	Hb (g/dL)	LY (K/uL)	Polys (K/uL)	Mono (K/uL)
4369_QN thymocyte_R1	190.520	469	7.7	110.54	51.40	20.8
4369_QN thymocyte_R2	High†	597	6.7	71.71	157.40	High
4369_QN thymocyte_R3	FD	FD	FD	FD	FD	FD
4369_QN thymocyte_R4	208.800	598	6.1	61	18	20
4369_QN thymocyte_R5	110.7	571	10.2	67.57	27.44	13.30

FD (Found dead).
QN (Quadruple Negative Cells).
† *High (out of range of the instrument and reported as "High").*

Supplementary Table 7. CBC from QN cells transplant recipients

NP23-NHD13 Donor	CBC					
	WBC (K/uL)	PLT (K/uL)	Hb (g/dL)	LY (K/uL)	Polys (K/uL)	Mono (K/uL)
QN thymocyte_100 cells_R1	181.06	753	10.70	27.81	114	22.25
QN thymocyte_100 cells_R2	162.14	305	5.40	28.15	96.77	26.61
QN thymocyte_100 cells_R3	144.62	415	10.20	42.11	70.05	21.26
QN thymocyte_100 cells_R4	161.50	346	8.60	20.70	97.51	20.70

Supplementary Table 8. CBC from kit-OP9 and Mac1$^+$/Gr1$^+$-OP9 population transplant recipients

NP23-NHD13 Donor Kit$^+$-OP9	CBC(at time of death)				
	WBC (K/uL)	PLT (K/uL)	Hb (g/dL)	LY (K/uL)	Polys (K/uL)
Recipients-2	6.97	401	5.1	5.6	0.27
Recipients-3	5.38	161	7.70	3.93	0.95
Recipients-4	6.96	170	9.8	5.31	1.29
Recipients-5	2.28	128	8.1	1.93	0.26

NP23-NHD13 Donor Mac-1$^+$/Gr-1$^+$-OP9	CBC(at time of death)				
	WBC (K/uL)	PLT (K/uL)	Hb (g/dL)	LY (K/uL)	Polys (K/uL)
Recipients-1	4.32	224	8.40	3.17	0.78
Recipients-2	118.20	218	9.20	20.78	72.44
Recipients-3	7.44	453	11.70	4.44	2.33
Recipients-4	2.68	137	2.30	1.06	1.45
Recipients-5	1.42	261	7.50	1.07	0.29

Supplementary Table 9. CBC from DN subpopulation transplant recipients

WT-Donor (DN cells)	CBC (10th Week)					
	WBC (K/uL)	PLT (K/uL)	Hb (g/dL)	LY (K/uL)	Polys (K/uL)	Mono (K/uL)
WT-Recipient-1	7.06	490	11.6	5.39	0.85	0.55
WT-Recipient-2	9.12	506	15.2	6.59	2.08	0.4
WT-Recipient-3	8.46	639	14.10	5.85	2.09	0.42
WT-Recipient-4	ND	ND	ND	ND	ND	ND
WT-Recipient-5	7.74	665	15.00	5.29	2.06	0.37

NP23-NHD13-Donor (DN1 cells)	CBC (10th Week)					
	WBC (K/uL)	PLT (K/uL)	Hb (g/dL)	LY (K/uL)	Polys (K/uL)	Mono (K/uL)
DN1-Recipient-2	248.36	469	5.10	94.56	113.10	30.82
DN1-Recipient-3	7.24	747	13.50	4.81	1.92	0.40
DN1-Recipient-4	6.70	147	6.80	5.36	0.87	0.38
DN1-Recipient-5	9.76	128	8.80	7.59	1.54	0.39

NP23-NHD13-Donor (DN2 cells)	CBC (10th Week)					
	WBC (K/uL)	PLT (K/uL)	Hb (g/dL)	LY (K/uL)	Polys (K/uL)	Mono (K/uL)
DN2-Recipient-1	3.18	295	9.60	2.43	0.51	0.23
DN2-Recipient-2	4.92	401	11.10	3.19	1.41	0.27
DN2-Recipient-3	6.00	309	11.50	4.08	1.60	0.28
DN2-Recipient-4	5.64	761	12.90	3.85	1.32	0.15
DN2-Recipient-5	15.64	163	7.40	12.40	2.10	1.00

NP23-NHD13-Donor (DN3 cells)	CBC(10th Week)					
	WBC (K/uL)	PLT (K/uL)	Hb (g/dL)	LY (K/uL)	Polys (K/uL)	Mono (K/uL)
DN3-Recipient-1	9.22	471	12.50	6.71	2.20	0.28
DN3-Recipient-2	7.66	638	12.00	5.07	2.08	0.25
DN3-Recipient-3	8.34	698	13.10	6.18	1.81	0.30
DN3-Recipient-4	9.04	748	14.40	6.78	1.54	0.55
DN3-Recipient-5	8.84	729	13.90	6.67	1.64	0.49

NP23-NHD13-Donor (DN4 cells)	CBC(10th Week)					
	WBC (K/uL)	PLT (K/uL)	Hb (g/dL)	LY (K/uL)	Polys (K/uL)	Mono (K/uL)
DN4-Recipient-1	8.54	629	12.80	6.04	2.17	0.28
DN4-Recipient-2	11.24	662	12.70	7.57	3.08	0.39
DN4-Recipient-3	7.06	722	13.50	5.01	1.66	0.30
DN4-Recipient-4	9.76	747	12.90	6.35	2.93	0.38
DN4-Recipient-5	8.06	473	10.60	6.77	0.81	0.41

NP23- NHD13-Donor (DN1 & DN2)	CBC(at time of death)					
	WBC (K/uL)	PLT (K/uL)	Hb (g/dL)	LY (K/uL)	Polys (K/uL)	Mono (K/uL)
DN1-Recipient-2	248.36	469	5.10	94.56	113.10	30.82
DN1-Recipient-5	10.56	60	3.3	9.29	0.34	0.15
DN2-Recipient-2	28.6	173	7.6	22.88	3.21	1.37
DN2-Recipient-3	5.06	230	10.40	4.02	0.54	0.10
DN2-Recipient-5	25.56	168	8.30	12.02	8.51	4.29

Not determined is indicated as "ND".

Supplementary Table 10. CBC from DN1 subpopulation transplant recipients

NP23-NHD13 Donor (DN1a cells)	CBC (10th Week)					
	WBC (K/uL)	PLT (K/uL)	Hb (g/dL)	LY (K/uL)	Polys (K/uL)	Mono (K/uL)
DN1a-Recipient-1	6.40	365	8.20	5.26	0.45	0.66
DN1a-Recipient-2	7.92	811	11.00	4.89	2.24	0.74
DN1a-Recipient-4	10.32	970	11.30	5.15	4.58	0.42
DN1a-Recipient-5	FD	FD	FD	FD	FD	FD

NP23-NHD13 Donor (DN1b cells)	CBC (10th Week)					
	WBC (K/uL)	PLT (K/uL)	Hb (g/dL)	LY (K/uL)	Polys (K/uL)	Mono (K/uL)
DN1b-Recipient-1	7.16	573	8.70	5.73	0.76	0.60
DN1b-Recipient-2	9.50	249	10.90	6.53	2.32	0.61
DN1b-Recipient-3	FD	FD	FD	FD	FD	FD
DN1b-Recipient-4	4.86	158	7.50	4.00	0.42	0.39

NP23-NHD13 Donor (DN1c cells)	CBC(10th Week)					
	WBC (K/uL)	PLT (K/uL)	Hb (g/dL)	LY (K/uL)	Polys (K/uL)	Mono (K/uL)
DN1c-Recipient-1	9.06	623	13.60	6.27	2.01	0.66
DN1c-Recipient-2	8.76	929	13.00	6.12	1.95	0.65
DN1c-Recipient-3	9.76	919	11.90	6.83	1.97	0.91
DN1c-Recipient-4	10.96	937	12.70	6.42	3.61	0.80
DN1c-Recipient-5	9.26	834	11.50	5.79	2.92	0.42

NP23-NHD13 Donor (DN1d cells)	CBC(10th Week)					
	WBC (K/uL)	PLT (K/uL)	Hb (g/dL)	LY (K/uL)	Polys (K/uL)	Mono (K/uL)
DN1d-Recipient-1	7.86	890	14.40	4.76	2.58	0.29
DN1d-Recipient-2	10.90	1060	12.00	8.10	2.13	0.59
DN1d-Recipient-3	8.08	752	13.20	6.00	1.49	0.55
DN1d-Recipient-4	6.52	753	12.80	4.54	1.54	0.39

NP23- NHD13- Donor (DN1a & DN1b)	CBC(at time of death)					
	WBC (K/uL)	PLT (K/uL)	Hb (g/dL)	LY (K/uL)	Polys (K/uL)	Mono (K/uL)
DN1a-Recipient-1	26.86	231	8.50	15.44	9.92	0.78
DN1b-Recipient-1	128.30	330	3.0	91.99	8.4	4.7
DN1b-Recipient-4	15.32	98	4.10	8.24	5.57	1.12

Found dead is indicated as "FD".

Supplementary Table 11. CBC from NP23-NHD13(LSK) transplant recipients

Donor WT-BM (LSK)	CBC (10th Week)					
	WBC (K/uL)	PLT (K/uL)	Hb (g/dL)	LY (K/uL)	Polys (K/uL)	Mono (K/uL)
Recipient-1	9.34	833	14.00	5.49	3.11	0.72
Recipient-2	11.00	988	13.00	5.91	5.16	0.61
Recipient-3	13.10	803	13.10	8.39	3.86	0.79

Donor NP23/NHD13-BM (LSK)	CBC (10th Week)					
	WBC (K/uL)	PLT (K/uL)	Hb (g/dL)	LY (K/uL)	Polys (K/uL)	Mono (K/uL)
Recipient-1	13.58	212	7.70	10.20	2.22	0.78
Recipient-2	7.10	250	11.70	4.59	1.96	0.53
Recipient-3	4.76	384	10.10	3.30	1.06	0.33
Recipient-4	ND	ND	ND	ND	ND	ND

Donor NP23/NHD13-Thymus(LSK)	CBC (10th Week)					
	WBC (K/uL)	PLT (K/uL)	Hb (g/dL)	LY (K/uL)	Polys (K/uL)	Mono (K/uL)
Recipient-1	7.64	621	13.30	4.73	2.41	0.47
Recipient-2	7.28	231	8.50	4.93	1.86	0.39
Recipient-3	11.02	539	12.70	6.95	3.31	0.64
Recipient-5	6.16	248	8.90	4.02	1.49	0.56

Supplementary Table 12. Primers used for different experiments

Primer name	Sequence 5'-3'	Gene
NUPPHF(F)	ATTTAATACTACGACAGCCACTTTGG	NUP98
PHF23 (R1)	CATCCAGATCAAAGAGAGAGTCC	PHF23
HOXD13-L1(F)	GGCTTCTAAGCTGTCTGTGGCC	HOXD13
NUP98001(R)	TGGAGGGCCTCTTGGTACAGG	NUP98
5'TCRBD1.1(F)	CTTATCTGGTGGTTTCTTCCAGC	TCRBD1
3'TCRBJ1S4.1(R)	CAGACAGCTTGGTTCCATGACCG	TCRBJ1S4
3'TCRBJ1S6.1(R)	AGACCATGGTCATCCAACACAGGC	TCRBJ1S6
5'TCRBD2.2(F)	TGTATCACGATGTAACATTGTGG	TCRBD2
3'TCRBJ2S4.2(R)	TACTGGGTGTCTTGGTTCACAGC	TCRBJ2
3'TCRBJ2S7.2 (R)	TTGAGAGCTGTCTCCTACTATCG	TCRBJ2S7
TCRVB(F)	TAAGCGGCCGCATGKDYTGGTAYMRRCAG	TCRVB
TCRCB(R)	CCCACCAGCTCAGCTCCACGTGG	TCRCB
SCIDA(F)	GGAAGAGTTTTGAGCAGACAATG	SCID
SCIDB(R)	CATCACAAGTTATAACAGCTGGG	SCID
